# Systematic review of the correlates of outdoor play and time among children aged 3-12 years

**DOI:** 10.1186/s12966-021-01097-9

**Published:** 2021-03-18

**Authors:** Eun-Young Lee, Ajaypal Bains, Stephen Hunter, Alyssa Ament, Javier Brazo-Sayavera, Valerie Carson, Shawn Hakimi, Wendy Y. Huang, Ian Janssen, Mikyung Lee, Heejun Lim, Diego Augusto Santos Silva, Mark S. Tremblay

**Affiliations:** 1 School of Kinesiology and Health Studies, Queen’s University, Kingston, ON Canada; 2 Department of Gender Studies, Queen’s University, Kingston, ON Canada; 3 Faculty of Kinesiology, Sport, and Recreation, University of Alberta, Edmonton, Canada; 4 Centro Universitario Regional Noreste, Universidad de la República, Rivera, Uruguay; 5 Centre for Health and Exercise Science Research, Department of Sport, Physical Education and Health, Hong Kong Baptist University, Hong Kong, China; 6 Department of Health Sciences, Queen’s University, Kingston, Canada; 7 Sports Centre, Federal University of Santa Catarina, Florianopolis, Brazil; 8 Healthy Active Lifestyle and Obesity (HALO) research group, Children’s Hospital of Eastern Ontario Research Institute, Ottawa, Canada

**Keywords:** Outdoor, Physical activity, Parent, Family, Socio-ecological modelling

## Abstract

**Background:**

Due to the myriad of benefits of children’s outdoor play and time, there is increasing concern over its decline. This systematic review synthesized evidence on the correlates of outdoor play and outdoor time among children aged 3-12 years.

**Methods:**

A total of 12 electronic databases in five different languages (Chinese, English, Korean, Spanish, Portuguese) were searched between October 28, 2019 and July 27, 2020. Covidence software was used for screening and Microsoft Excel with a predesigned coding form was used for data extraction. Evidence was synthesized and correlates were categorized using the socioecological model framework.

**Results:**

Based on 107 studies representing 188,498 participants and 422 childcare centers from 29 countries, 85 studies examined potential correlates of outdoor play while 23 studies examined that of outdoor time (one examined both). The duration of outdoor play and outdoor time ranged between 60 and 165 min/d and 42-240 min/d, respectively. Out of 287 (outdoor play) and 61 (outdoor time) potential correlates examined, 111 correlates for outdoor play and 33 correlates for outdoor time were identified as significant correlates. Thirty-three variables were identified as key/common correlates of outdoor play/time, including eight correlates at the individual level (e.g., sex/gender, race/ethnicity, physical activity), 10 correlates at the parental level (e.g., parental attitude/support/behavior, parenting practice), nine at the microsystem level (e.g., proximal home/social environment such as residence type, peer influence), three at the macrosystem/community level (e.g., availability of space children can play), and three at the physical ecology/pressure for macrosystem change level (e.g., seasonality, rurality). No key correlates were found at the institutional level.

**Conclusions:**

Individual, parental, and proximal physical (home) and social environments appear to play a role in children’s outdoor play and time. Ecological factors (i.e., seasonality, rurality) also appear to be related to outdoor play/time. Evidence was either inconsistent or lacking at institutional and macrosystem/community levels. Standardizing terminology and measures of outdoor play/time is warranted. Future work should investigate the interactions and processes of multiple variables across different levels of socioecological modelling to better understand the mechanisms through which outdoor play/time opportunities can be optimized for children while paying special attention to varying conditions in which children are born, live, and play.

**Supplementary Information:**

The online version contains supplementary material available at 10.1186/s12966-021-01097-9.

## Introduction

Outdoor play or simply spending time outdoors is beneficial for healthy growth and development among children [1–3]. Though ambiguity exists in terminology, playing or spending time outdoors, commonly operationalized as ‘outdoor play’ or ‘outdoor time’ (outdoor play/time hereafter), is a main source of moderate- to vigorous-intensity physical activity (MVPA) [4–6]. Building on the emerging time-use epidemiology pertaining to 24-h movement behaviors (i.e., physical activity, sedentary behavior, sleep), replacing indoor time with outdoor time can help children to accumulate more MVPA and thus gain additional health benefits [7–9].

Despite the known benefits of outdoor play/time to children’s health, evidence suggests that outdoor play/time has been decreasing over the years [3]. There are potentially multiple layers of influence on such decrease, including lifestyle changes [10] due to urbanization [11] and technological advancement [12], children’s safety and parental concerns [13, 14], and changing social norms around children’s independent mobility [3, 15]. Opportunities for children to engage with outdoor, natural environments may continue to decrease in a constantly evolving socio-environmental world. This prospect became realized with our current experience of the COVID-19 pandemic, where the mass home-confinement directives and restrictions on the use of public outdoor spaces are deterring outdoor play among children [16–18].

According to the behavioral epidemiology framework, identifying correlates of health behavior is critical for developing and refining successful behavior change interventions for population health [19]. Two recent systematic reviews [20, 21] have sought to identify important correlates of outdoor play. These reviews suggested a number of parental and built environmental correlates, including mother’s ethnicity and employment status [20], high parental education [20], social cohesion [20], low traffic volumes [21], access to a yard [21], and high neighborhood greenness [21]. These findings can serve as a groundwork for better understanding of the correlates and developing intervention programs to increase outdoor play among children; however, some gaps are also noted. Specifically, Boxberger and Reimers [20] only focused on perceived parental correlates of children’s outdoor play. While Lambert and colleagues [21] included both device-based and subjective correlates, their review exclusively focused on the influence of neighborhood built environment on outdoor play. In one review, only outdoor play was operationalized [20] without making clear distinctions between outdoor play and outdoor time. Lambert and colleagues [21] had an exclusive focus on outdoor play; nevertheless, they provided a definition of play, which is “freely chosen, personally directed, intrinsically motivated behavior that actively engages the child [22].”

Socioecological modelling (SEM) [23, 24] acknowledges that there is a myriad of factors embedded within several levels of influences (e.g., interpersonal, institutional, societal) that act and interact to shape behavior. Building on the two previous reviews that had an exclusive focus on outdoor play only and operationalized only two levels of influence (i.e., parental, built environment) within the SEM framework [20, 21], the purpose of the current systematic review was to synthesize the literature on the correlates of outdoor play/time, inclusively, among children aged 3-12 years using a broad, multi-factorial SEM framework [23, 24] and comprehensive, multilingual search strategy. Our goal was to gain a more comprehensive understanding of the factors that may facilitate or inhibit children playing or spending time outdoors.

## Methods

This systematic review used the Preferred Reporting Items for Systematic reviews and Meta-Analysis (PRISMA) guidelines as a guiding framework [25]. The review protocol was registered on PROSPERO (PROSPERO 2020 CRD42020152469), the international prospective register of systematic reviews (https://www.crd.york.ac.uk/prospero/display_record.php?RecordID=152469). For the purpose of this review that summarizes correlates of outdoor play and outdoor time, separately and together, outdoor play refers to the duration, intensity, volume, and/or frequency of free, unstructured play outdoors. Both inactive and physically active play were considered inclusively. Outdoor time refers to the duration and/or frequency of time spent outside.

### Eligibility criteria

To be eligible for this review, a study had to meet the following criteria: a) includes children aged between 3 and 12 years, b) reports a quantitative measure of outdoor time/play (subjective or objective), c) measures an association with at least one correlate (exposure/independent variable) and outdoor time/play (outcome), c) uses cross-sectional, case-control, cohort, intervention study design, d) published in peer-reviewed journal in the year 2000 and onward to only capture recent publications, and e) have an analytic sample of at least 100 participants to ensure that all results that are included in this review are based on sufficient statistical power. Case studies and qualitative studies were excluded. Alterative terms related to outdoor play (e.g., outdoor free play, outdoor unstructured play, active outdoor play, play outside, outdoor playtime) or outdoor time (e.g., time spent outside, outside time) identified from our searches were considered for inclusion as long as an article included the term related to being outdoors (e.g., out, outside, outdoor) in addition to “play” or “time”; however, indoor play/time or not specified was deemed to be ineligible. Furthermore, if a study specifically measured physical activity at different intensity rather than “play” or “time spent” per se, it was deemed to be ineligible. Studies limited to children with a known health or behavior condition (with the exception of overweight/obesity) were excluded.

### Information source and search strategy

Literature searches were conducted in five different languages. These languages were selected primarily based on the languages spoken by the co-authors. For English articles, MEDLINE, PsycINFO, SPORTDiscus, Sports Medicine & Education Index, CINAHL, and Web of Science were searched (EL and MC). For Chinese Mandarin (Chinese thereafter), CNKI (China National Knowledge Infrastructure) and WanFang Data were searched (WYH). For Korean, KISS (Korean Information Service System) was searched (EL). For Portuguese, SciELO (Scientific Electronic Library Online) and LILACS (Latin American and Caribbean Health Science Literature) were used (DASS). For Spanish, MEDLINE in Spanish, Latindex, LILACS, and SCIELO were searched (JB-S and BBP). Keywords and search strings for each database are presented in Supplementary Table 1. The initial English search strategy was developed by the primary investigator (EL) in collaboration with a research librarian (MC). The searches were restricted by English language and human participants for English databases and human participants only for other languages. Search strategies for other languages were developed by DASS, EL, JB-S, and WYH in their respective languages based on the English version. Specific information on search strategy by each language is described in Supplementary Table 1. The searches were first done between October 28 to November 4, 2019 and top-up searches were conducted on July 27, 2020 for English articles. Searches in Chinese, Korean, Portuguese, and Spanish were conducted between June 1, 2020 to June 23, 2020. For English articles, the final search results in each database were imported into the Clarivate Analytics EndNote X9 then Covidence (www.covidence.org)—a web-based software for screening selected data. For other languages, Microsoft Excel was used. Hand-searching by the primary investigator (EL) was also conducted on November 5, 2020 to ensure that the most up-to-date, relevant studies post top-up search (July 27, 2020) were also included in the review.

### Study selection

Best practice guidelines for abstract screening large-evidence systematic reviews and meta-analysis outlined by Polanin and colleagues [26] were followed for the Level 1 screening (title and abstract). Briefly, it consisted of the following 10 steps for the screening of titles and abstracts of identified studies from (1) creating a clear and concise abstract screening tool, (2) ensuring the hierarchical organization of the abstract screening tool, (3) conducting introductory abstract screening, (4) meeting with the screening team on a bi-weekly basis, (5) minimizing changes to the screening tool, (6) using a text-mining abstract screening application, (7) conducting independent double-screening of each study, (8) resolving conflicts, (9) encouraging screening through incentives, and (10) analyzing the process and decisions after the completion of the screening. For all languages, double screening was used at both Level 1 and Level 2 (full text) (Screeners for English articles, *n* = 7; screeners for articles in other languages, *n* = 2 for each language). Any disagreement was resolved through a consensus discussion and if consensus could not be reached the final inclusion of articles was decided by a third reviewer. In cases where a decision for exclusion or potential inclusion could not be made by the title/abstract, the full text was retrieved. At Level 1, disagreement reconciliation occurred after every third of the abstracts had been screened [26]. Different numbers of screeners were involved for each language with varying inter-rater reliability, which are described in Supplementary Table 2. Overall, inter-rater reliability (Cohen’s κ ) ranged between moderate (0.41) and almost perfect (0.94).

### Data collection process and data items

Data extraction was conducted in the Microsoft Excel spreadsheet developed by the primary investigator (EL). Bibliographic information (i.e., authors and year of publication), setting and study design; sample characteristics (sample size, mean age, sex-male and female (n and %), exposure and outcome measurements, and potential correlates of outdoor play and relevant statistics were extracted. Six extractors (AB, EL, HL, ML S Hakimi, and S Hunter) were paired for English articles with one researcher extracting data from assigned articles then extracted data were reviewed and verified by another researcher. For other languages, two extractors conducted data extraction for each language (Chinese: WYH and JJF; Spanish: JB-S and BBP; Portuguese: DASS and GC). Discrepancies were resolved through consensus discussion. Remaining disagreements were resolved through discussions with the primary investigator (EL).

### Risk of bias assessment

The modified Cochrane Collaboration tool [27] in the Cochrane Handbook (http://handbook.cochrane.org/) was used to assess risk of bias for included studies. Bias was assessed as a judgement (high, low, or unclear) for the following six domains: (1) selection, (2) performance, (3) detection, (4) attrition, (5) reporting, and (6) other. The tool included core elements of appropriate selection of participants (inadequate randomization and allocation concealment for intervention studies and flawed method of participant selection for observational studies), measurement of exposure (knowledge of allocated intervention studies during the study for intervention studies and acceptable reported measurement details of the proposed correlates for observational studies), measurement of outcome (knowledge of outcome assessors for intervention studies and flawed measurement of outcome or differential misclassification for observational studies), attrition (amount, nature, or handling of incomplete outcome data for intervention studies, incomplete/high loss to follow-up or missing data for observational studies), reporting (selective outcome reporting for both intervention and observational studies), and other sources of bias (bias due to problems not covered elsewhere in a study). The six criteria were judged with either “low (1 point)”, “high (0 points)”, or “unclear (0 points)”. High quality (low risk of bias) was considered a score of five or six, moderate quality was considered with scores of three or four, and low quality (high risk of bias) was considered with scores of zero to two. Risk of bias assessment was undertaken by pairs of extractors and discrepancies were addressed through discussion in pairs. A third independent reviewer was introduced when discrepancies could not be resolved.

### Analysis and synthesis of results

Meta-analyses were planned but not conducted due to heterogeneity of the data which could not be meaningfully pooled (i.e., if data were too diverse in terms of statistical, clinical, and methodological characteristics). Thus, narrative syntheses of research findings were conducted to identify potential correlates of outdoor play/time. Potential correlates of outdoor play/time were grouped into six different levels informed by SEM [23, 24]: (1) individual (i.e., children’s characteristics), (2) parental (i.e., parental characteristics), (3) microsystem (i.e., immediate setting where children interact with their parents/guardians and siblings), (4) institutional (i.e., physical and social microenvironments such as childcare or school), (5) macrosystem/community (i.e., distal physical and sociocultural environments such as the built environment), and (6) physical ecology/pressure for macrosystem change (i.e., the most distal level of influence such as urbanization, climate). The direction of the association between each correlate investigated and outdoor play/time was indicated as positive (+), negative (−), or null (∅). Statistically adjusted findings for varying covariates were preferred but unadjusted findings were used when adjusted findings were not available. All statistical techniques were considered; however, outdoor play/time entered as independent or predictor variable in directional statistical techniques (e.g., t-tests, linear or logistic regression analysis) were deemed to be ineligible and excluded from synthesis. If experimental design was used, only baseline characteristics were considered. Only statistically significant results based on hypothesis testing with alpha level < 0.05 were considered in determining important correlates.

Similar to previous reviews [20, 28, 29], the consistency of association of each of the potential correlates were determined based on the percentage of reported findings that support the hypothesized association. The hypothesized association was measured by dividing the number of observations supporting the association by the total number of observations where the association was investigated. When the results varied by subgroups (e.g., younger age/older age, boy/girl, weekday/weekend, urban/rural), findings were reported separately to account for varying results based on observations stratified by subgroups. Percentages ranging between 0 and 33% were considered as ‘no evidence (coded as “Ø”)’, 34–59% as ‘inconsistent evidence (coded as “?”)’ with the most frequent direction of the association reported (coded as “ + ” or “ − ” based on consistent direction of the association), and 60–100% as ‘consistent evidence (coded as “ + ” or “ − ” based on consistent direction of the association)’. To indicate the strength of evidence, the result was coded as ‘ ØØ,’ ‘ ++,’ or ‘− − ’ when ≥ four observations were observed; a single symbol was used if there were three or fewer observations. Reporting was stratified by age, sex/gender, and weekday/weekend if directions were inconsistent across the categories of those variables; consistent direction was reported only once to avoid drawing strong evidence from one study only. Correlates of outdoor play/time were further synthesized by identifying *key*, *common*, and *consistent* correlates that were not mutually exclusive. To be considered as *key* correlates, the evidence had to be based on at least two observations. Among those, *common* correlates indicated correlates identified for both outdoor play and time. *Consistent* correlates included correlates that showed consistent associations (≥ 60% of at least four observations). For example, ‘age’ could be a *key* correlate for outdoor play and time which makes ‘age’ a *common* correlate. Also, if ‘age’ is supported as a *key* correlate for outdoor play in more than 60% of the evidence, it is also considered as a *consistent* correlate.

All studies, regardless of the quality rating, were included in analyses and discussing the overall review findings and for sensitivity analyses. Subgroup analyses were planned if sufficient data were available by age, sex/gender, self-report vs direct measure of outdoor play/time, type of outdoor activities (e.g., outdoor play, outdoor time), season/climate, urbanicity vs rurality, and country or region of studies. However, only pooled results were reported because of heterogeneity across studies.

## Results

### Study selection

**Fig. 1:**
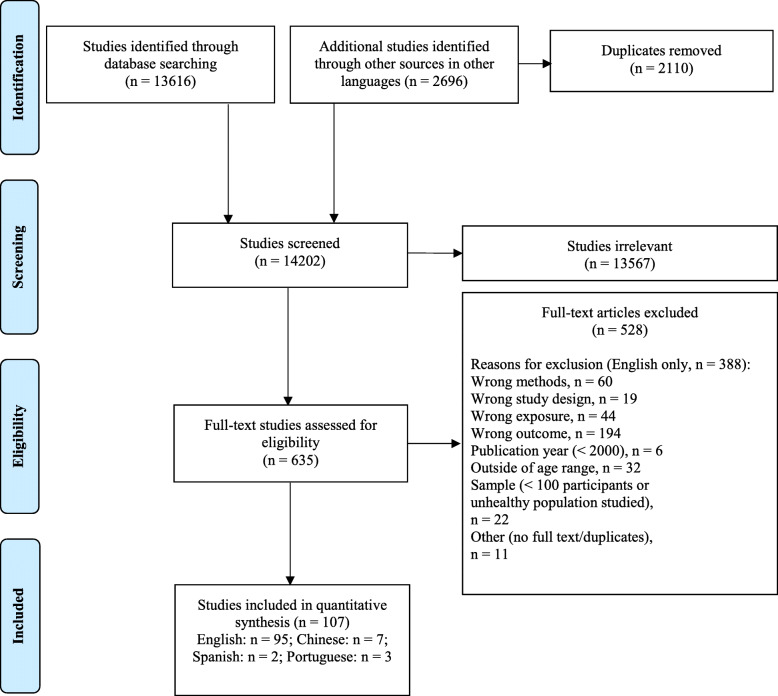
PRISMA flow diagram

The number of studies included in the title and abstract screening and full text screening by language are provided in detail in Supplementary Table 2 and the overall PRISMA flowchart for study selection is described in Fig. 1. A total of 13,616 studies in English and 2696 studies in other languages were imported. After removing 2110 duplicates, 14,202 studies were assessed for eligibility with title and abstract screening. Of these, 13,567 studies were excluded leaving 635 studies for full-text screening. After removing 528 irrelevant studies and adding one relevant study using hand-searching, 107 studies were included in this review. Twenty-four unique intervention studies were identified; however, 22 of these were excluded because no relevant baseline data were provided. The two intervention studies [30, 31] that were included provided baseline observational data and were coded as cross-sectional studies for our data synthesis.

### Study characteristics

Descriptive characteristics of the 107 studies are described in Table 1. These studies represented a total of 188,498 unique participants and one study involving 422 Early Childhood Education and Care (ECEC) centers [70, 105], with the analytic samples ranging between 100 and 29,159 and from 29 countries. Of 107 studies included, 27 studies were based on samples from the US [45, 46, 57, 62, 63, 66–69, 72, 77, 81–83, 87, 92, 93, 96, 102, 106, 118, 119, 121, 125, 126, 132], followed by Australia (*n* = 14) [31, 36, 50, 51, 53, 54, 65, 105, 114, 116, 122, 129–131] and Canada (*n* = 11) [50, 60, 61, 73, 76, 98, 105, 115, 117, 135] (one study included both Australia and Canada [70]). There were 96 cross-sectional [31–35, 38, 40–50, 52–56, 58–102, 104–110, 113–121, 123–130, 134–137] and 12 longitudinal studies (prospective, n = 11 [39, 53, 54, 103, 111, 112, 130, 133, 137]; retrospective, n = 1 [65]), of which two studies included both cross-sectional and longitudinal designs [37, 96]. Among 88 studies that reported sex/gender distribution of their sample [32–35, 37, 38, 40, 42, 44–49, 51–57, 59–62, 64–78, 82–85, 87–90, 92, 93, 95–104, 106–126, 128–135, 138], one was limited to girls in the UK [35] and in the remaining 87 studies an average of 49.6% were girls. Age of participants was reported as mean (7.7 years; *n* = 58 studies) or ranged between 0 and 14 years (*n* = 46 studies). In four studies school grade was reported (1st grade, *n* = 1; 5th grade, *n* = 2, pre-kindergarten to 6th grade, *n* = 1) [47, 59, 102, 113, 132]. The quality rating scores are presented in Supplementary Table 3. Quality was rated as high in 28 studies (25.9%) [35, 39, 40, 46, 48, 53, 54, 59, 61, 65, 67–70, 74, 80, 82, 87, 92, 101–103, 107, 115, 120, 121, 130, 135], moderate in 60 studies (56.1%) [30, 32–37, 40, 41, 43–48, 52, 55, 56, 61–65, 70, 72–75, 77, 78, 82, 84, 86, 88–91, 94, 99, 101, 103, 106, 108–112, 114–118, 120, 122, 124, 127, 128, 132, 133, 135], and low in 20 studies (18.7%) [33, 34, 36, 43, 52, 60, 62, 64, 84, 86, 93, 96, 97, 99, 108, 124–126, 129, 131, 132, 137].

**Table 1:** Descriptive characteristics of the included studies (in an alphabetical order of the last name of first author) (*N* = 107 studies)

First author (year)	Language	Analytic sample (n)	Female/girl (%)	Participant age^a^	Country	Study design	Duration/frequency of outdoor play or outdoor time reported
Aarts (2010) [32]	English	6470	49.9%	4-12 years	Denmark	Cross-sectional	Outdoor play duration Total: 6.9 h/wk 4-6 years: Boys 417 min/wk, Girls 390 min/wk 7-9 years: Boys 449 min/wk, Girls 396 min/wk 10-12 years: Boys 443 min/wk, Girls 373 min/wk
Aarts (2012) [33]	English	3651	49.0%	7.8 years old	Netherlands	Cross-sectional	Outdoor play duration Total: 6.9 h/wk 4-6 years: Boys 408 min/wk, Girls 378 min/wk 7-9 years: Boys 455 min/wk, Girls 398 min/wk 10-12 years: Boys 444 min/wk, Girls 381 min/wk
Aggio (2017) [34]	English	13,169	49.3%	7.2 (0.3) years old	UK	Cross-sectional	Outdoor play (independent) frequency 29% engaged in independent outdoor play
Anthamatten (2014) [35]	English	9900	48.5%	5-12 years	USA	Cross-sectional	NR
Armstrong (2019) [36]	English	224	46.0%	2.7 (0.7) years old	Australia	Cross-sectional	Outdoor play duration 68.9 (2.2) min/d in the home yard
Bagordo (2017) [37]	English	1164	48.1%	7.3 (0.9) years old	Italy	Cross-sectional and Longitudinal-Prospective cohort	Outdoor play duration (% of children with ≥ 1 h/d) Season I (winter): 33.0% Season II (late spring): 70.3%
Barros (2012) [38]	Portuguese	265	46.0%	Girls: 5.0 (0.9) years oldBoys: 4.8 (0.8) years old	Brazil	Cross-sectional	Outdoor play duration (% of children with ≥ 1 h/d) 65.3%
Barros (2019) [39]	Portuguese	700	NR	4.8 (0.8) years old	Brazil	Longitudinal-Prospective cohort	NR
Berger (2019) [39]	English	1160	42.9%	11-12 years at baseline	England	Longitudinal-Prospective cohort	Outdoor play frequency Total: 75.3% White: 71.1% White mixed: 75.1% Bangladeshi: 74.8% Black African: 80.1%
Berglind (2017) [40]	English	899	NR	4.0 years old	Sweden	Cross-sectional	Outdoor play duration Total: 136.4 min/d Weekday: 90.1 min/d Weekend: 182.6 min/d Outside of preschool hours Girls Weekday: 85.1 min/d Weekend: 174.7 min/d Boys Weekday: 95.9 min/d Weekend: 189.3 min/d
Bohn-Goldbaum (2013)^b^ [30]	English	164 for direct observation; 140 for interview	NR	2-12 years for direct observation; ≥ 16 years+ with children under 13 years for interview	Australia	Cross-sectional	NR
Boldemann (2006) [41]	English	199	42.7%	4.5-6.5 years	Sweden	Cross-sectional	Outdoor time duration (≥ 1 h/d on an ordinary Sunday) 95%
Boldemann (2011) [42]	English	169	NR	3.0-5.9 years	Sweden	Cross-sectional	Outdoor time duration (≥ 1 h/d on an ordinary Sunday) 100%
Bourke (2014) [43]	English	1534	50.3%	0-14 years	New Zealand	Cross-sectional	NR
Bringolf-Isler (2010) [44]	English	680	48.8%	6-10 years	Switzerland	Cross-sectional	Outdoor play duration 81.8 min/d
Brown (2009) [45]	English	372	49.0%	3-5 years	USA	Cross-sectional	NR
Burdette (2005) [46]	English	3141	47.0%	39.0 (3.0) months old	USA	Cross-sectional	Outdoor play duration Total: 191 min/d Weekday: 156.0 (120.0) min/d Weekend: 226.0 (140.0) min/d
Burgi (2016) [47]	English	209	42.6%	2nd grade: 8.5 (0.3) years old6th grade: 12.5 (0.4) years old	Switzerland	Cross-sectional	Outdoor time duration (median) Total: 83.8 min/d Second grader Weekday: 83.6 min/d on street and 0.0 min/d outside Weekend: 92.4 min/d on street and 5.7 min/d outside Sixth grader Weekday: 78.0 min/d on street and 0.0 min/d outside Weekend: 81.2 min/d on street and 0.0 min/d outside
Cardon (2008) [48]	English	783	47.0%	5.3 (0.4) years old	Belgium	Cross-sectional	NR
Caroli (2011) [49]	English	1094	46.4%	56.6 (11.3) months old	DenmarkItalyPoland	Cross-sectional	Outdoor play duration (≥ 1 h/d) Weekday Denmark: 19.8% Italy: 22.2% Poland: 35.0%
							Weekend Denmark: 86.7% Italy: 54.4% Poland: 91.1%
Carsley (2017) [50]	English	2810	48.4%	1-5 years	Canada	Cross-sectional	Outdoor play duration (median) Total: 60.0 min/d Attends daycare: 45.0 min/d Does not attend daycare: 60.0 min/d
Christian (2014) [51]	English	727	52.2%	11 (0.8) years old	Australia	Cross-sectional	Outdoor play frequency Total: 70.1% (In the yard: 87.6%; On street: 52.6%)
							Non-dog walkers In the yard: 84.3%; On street: 44.8%
							Dog walkers In the yard: 90.8%; On street: 60.3%
Christian (2019) [52]	English	678	47.0%	3.4 (0.8) years old	Switzerland	Cross-sectional	Outdoor time duration 187.6 min/d in ECEC
Cleland (2008) [53]	English	548	53.2%	5-12 years	Australia	Longitudinal-Prospective Cohort	Outdoor time duration Total at baseline: 6.2 h/wk
							Warmer months at baseline Weekday: 7.7 h/wk. Weekend: 9.4 h/wk
							Cooler months at baseline Weekday: 3.0 h/wk. Weekend: 4.8 h/wk
Cleland (2010) [54]	English	421	NR	5-6 years and 10-12 years	Australia	Longitudinal-Prospective Cohort	NR
Conrad (2013) [55]	English	1670	49.0%	3-14 years	Germany	Cross-sectional	Outdoor time duration 223 min/d
							> 2 h/d 5%
Cooper (2010) [56]	English	1010	NR	11.0 (0.4) years old	England	Cross-sectional	Outdoor time duration 41.7 (46.1) min/d outside after school each day (between 3:30 pm and 8:30 pm)
Damore (2002) [57]	English	254	NR	2-12 years	USA	Cross-sectional	Outdoor time duration Total ≥ 4 d/week: 69.8%
							Preschooler ≤ 3 d/week Suburban: 14%; Urban: 48% ≥ 4 d/week Suburban: 86%; Urban: 52%
							School-aged ≤ 3 d/week Suburban: 13%; Urban: 46% ≥ 4 d/week Suburban: 87%; Urban: 54%
							Among suburban children only (> 1 h/d) During school year: 23% During summer: 63%
Donatiello (2013) [58]	English	1673	48.1%	6.1 (1.7) years old	Italy	Cross-sectional	Outdoor play duration Total: 148.9 min/d
							Rural: 185.2 min/d Suburban:132.0 min/d Urban: 129.4 min/d
Dregval (2009) [59]	English	515	49.7%	1st grade student	Lithuania	Cross-sectional	Outdoor play duration (≥ 3 h/d) 36.9%
Faulkner (2015) [60]	English	859	52.5%	10.5 (0.7) years	Canada	Cross-sectional	Outdoor play duration (≥ 2 h/d) Total: 20.0% Weekday: 7.8%; weekend: 32.1%
Ferrao (2015) [61]	English	514	51.2%	7-12 years	Canada	Cross-sectional	Outdoor play frequency (daily) 13.3%
Frech (2011) [62]	English	3572	48.0%	61.7 (2.8) months old	USA	Cross-sectional	Outdoor play frequency (weekly) 3.8 days with mother
Galvez (2013) [63]	English	324	71.0%	6-8 years	USA	Cross-sectional	Outdoor play duration (median) 2 h/wk.
Gao (2017) [64]	Chinese	1689	NR	Primary school: 10.6 (0.9) years old High school: 13.5 (0.8) years old	China	Cross-sectional	Multiple groups, but no overall mean Mean score of outdoor play time (1: < 1 h; 2: 1 h; 3: 2 h; 4: 3 h; 5: > = 4 h) Boys (Primary): 12.6 ± 4.0 Boys (High school): 11.8 ± 4.1 Girls (Primary): 12.0 ± 3.7 Girls (High school): 11.0 ± 4.1
Gopinath (2013) [65]	English	1794	50.2%	12.7 years old	Australia	Longitudinal-Retrospective Cohort	Outdoor play duration (≥ 3710 h/wk) 25.1%
Gottfried (2017) [66]	English	14,370	49.0%	66.1 months old	USA	Cross-sectional	NR
Grigsby-Toussaint (2011) [67]	English	365	47.5%	2-5 years	USA	Cross-sectional	Outdoor play duration 60 min/d
Gross (2013) [68]	English	401	45.4%	5 years	USA	Cross-sectional	Outdoor play duration 60 min/d
Hammond (2011) [69]	English	140	42.1%	6-13 years	USA	Cross-sectional	NR
Hinkley (2015) [70]	English	Australia: 71 ECEC centres and 65 preschools; Canada: 46 ECEC centres	NR	3-5 years	AustraliaCanada	Cross-sectional	Outdoor time duration (in ECEC) Total: 125.3 min/d Australian ECEC: 143.8 min/d Canadian ECEC: 106.8 min/d
Hornby-Turner (2014) [71]	English	145	100.0%	White British: 9.9 (1.0) years old; British Pakistani: 10.0 (0.7) years old	UK	Cross-sectional	Outdoor play frequency (24-h recall) Total: 62.8% White British: 67.3% British Pakistani: 58.5%
Howie (2013) [72]	English	231	49.1%	4.3 years old	USA	Cross-sectional	NR
Hunter (2020) [73]	English	1212	48.3%	8.5 (3.6) years old	Canada	Cross-sectional	Outdoor time duration Total: 240 min/d Weekday: 240 min/d Weekend: 240 min/d
Imhof (2016) [74]	English	358	51.4%	7.3 (0.4) years old	Switzerland	Cross-sectional	Outdoor play duration 76 min/d
Islam (2016) [75]	English	109	38.5%	11.6 (1.2) years old	Bangladesh	Cross-sectional	Outdoor time frequency (during weekdays) 33.9%
Janssen (2015) [76]	English	724	49.5%	7-12 years	CanadaUSA	Cross-sectional	Outdoor play frequency 13.3 (12.6–14.0) times/wk
Jerrett (2013) [77]	English	147	52.5%	8-14 years	USA	Cross-sectional	NR
Jin MH (2020) [78]	Chinese	1473	NR	5.0 (1.1) years old	China	Cross-sectional	Outdoor play duration 46.1 min/d
Jin F (2020) [79]	Chinese	121	NR	4-6 years	China	Cross-sectional	NR
Jones (2009) [80]	English	100	53.0%	9-10 years	UK	Cross-sectional	NR
Kepper (2020) [81]	English	30 parents; 263 Adolescents	53.2%	12.5 (1.9) years old	USA	Cross-sectional	NR
Kimbro (2011) [82]	English	1975	49.0%	63.5 (3.0) months old	USA	Cross-sectional	Outdoor play duration Weekday: 126 min/d
Kneeshaw-Price (2013) [83]	English	682	49.9%	9.1 (1.6) years old	USA	Cross-sectional	NR
Kocken (2012) [84]	English	878	49.2%	6-10 years	Netherlands	Cross-sectional	NR
Kos (2013) [85]	English	68 teachers196 parents ofchildren	NR	3-6 years	Slovenia	Cross-sectional	Outdoor play duration Total: 8.4 h/wk
							Warm months: 10.6 h/wk. in playground or nature Cold months: 6.2 h/wk. in playground or nature
							Outdoor play frequency (% of the day consisting outdoor activities) 24.6% during warm months 14.6% during cold months
Lachowycz (2012) [86]	English	902	52.5%	11-12 years	UK	Cross-sectional	Outdoor play duration Total: 35.9 min/d
							Weekday evenings (3 pm-10 pm) Sedentary: 14.5 min/d; 7.0% Light: 9.1 min/d; 11.7% MVPA: 7.0 min/d; 26.4%
							Weekend days (8 am-10 pm) Sedentary: 20.7 min/d; 5.3% Light: 13.0 min/d; 8.5% MVPA: 7.5 min/d; 17.6%
Larson (2019) [87]	English	543	56.0%	12.9 (0.7) years old	USA	Cross-sectional	Outdoor time duration 102 min/d
Lee RE (2016) [88]	English	1321	52.6%	9.6 years old	Mexico	Cross-sectional	Outdoor play frequency 75.8% of children answered yes when asked if they played outside
Lee ST (2016) [89]	English	835	49.2%	5.4 (0.1) years old	Malaysia	Cross-sectional	NR
Liu MY (2020) [90]	Chinese	454	NR	30-85 months old	China	Cross-sectional	Outdoor play duration Total: 123 min/d Weekday: 90 min/d Weekend: 156 min/d
							Outdoor play frequency (≥ 4 times/week) Total: 49.3% K1: 26.6%; K2: 20.7%; K3: 2.0%
Liu TT (2020) [91]	Chinese	1506	NR	9.2 (2.1) years old	China	Cross-sectional	Outdoor play duration Total: Urban weekday: 4.8 (0.5) hours Urban weekend: 3.0 (1.1) hours Rural weekday: 8.1 (0.9) hours Rural weekend: 5.2 (1.5) hours
Marino (2012) [92]	English	878	50.8%	6-11 years	USA	Cross-sectional	NR
Martin (2008) [93]	English	331	55.5%	12.1 (1.0) years old	USA	Cross-sectional	NR
Martino (2019) [94]	English	29,159	50.0%	9.8 years old	Italy	Cross-sectional	NR
Matarma (2020) [95]	English	712	47.8%	5.1 (0.1) years old	Finland	Cross-sectional	Outdoor play duration (≥ 60 min/d) Total: 65.5% Boys: 70.2%; Girls: 60.8%
McHale (2001) [96]	English	198	51.5%	10.9 (0.5) years old	USA	Cross-sectional and Longitudinal-Prospective Cohort	NR
Miranda-Rios (2017) [97]	Spanish	192	42.2%	5-12 years	Mexico	Cross-sectional	NR
Moore (2020) [98]	English	1472	47.0%	45.1 (7.5) months old	Canada	Cross-sectional	Outdoor time duration Total: 143 min/d Girls: 141 min/d; Boys: 145 min/d
Moran (2017) [99]	English	573	50.0%	10-12 years	Israel	Cross-sectional	NR
Mota (2017) [100]	Portuguese	485	46.0%	Girls: 4.7 (0.9) years old Boys: 4.8 (0.9) years old	Portugal	Cross-sectional	Outdoor play duration Total: 148 min/d Weekday Girls: 90 min/d; Boys: 100 min/d Weekend Girls: 189 min/d; Boys: 211 min/d
Muthuri (2015) [101]	English	563	53.5%	9-11 years	Kenya	Cross-sectional	NR
Nicksic (2018) [102]	English	748	57.9%	5th grade students	USA	Cross-sectional	Outdoor play duration (≥ 30 min/d) Child reported: 3.2% Girls: 3.0%; Boys: 3.3% Parent reported: 82.2% Girls: 79.2%; Boys: 86.4%
Nordvall-Lassen (2018) [103]	English	4941	49.6%	9-11 years	Denmark	Longitudinal-Prospective Cohort	NR
Nystrom (2019) [98]	English	1699	55.0%	10.2 (1.0) years old	Canada	Cross-sectional	NR
Page (2010) [104]	English	1307	48.9%	10-11 years	UK	Cross-sectional	NR
Predy (2020) [105]	English	240 directors of ECEC (3 for toddler groups, 19 for preschool groups, and 218 for both)	NR	Toddlers: 19–35 months; Preschoolers: 36–60 months	Canada	Cross-sectional	Duration Toddler: Median 30-44 min/d during winter months Median 75-89 min/d during non-winter months Preschooler: Median 45-59 min/d during winter moths Median 90-119 min/d during non-winter months Frequency Toddler: 1 time/d during winter months 2 time/d during non-winter months Preschooler: 2 time/d during winter and non-winter months
Puett (2019) [106]	English	144	NR	9.6 (1.6) years old	USA	Cross-sectional	NR
Ramirez-Izcoa (2017) [107]	Spanish	357	53.5%	6-11 years	Honduras	Cross-sectional	Outdoor play duration > 60 min/d: 27.7%
Reimers (2017) [108]	English	2538	50.6%	0-12 years	Germany	Cross-sectional	NR
Reimers (2018) [109]	English	266	44.0%	4-12 years	Germany	Cross-sectional	NR
Reimers (2019) [110]	English	3505	51.0%	12.0 (3.3) years old	Germany	Cross-sectional	NR
Remmers, Broeren (2014) [111]	English	2007	49.5%	5.8 (0.4) years old at baseline	Netherlands	Longitudinal-Prospective Cohort	Outdoor play duration 109.0 min/d
Remmers, Kann (2014) [112]	English	1875	49.0%	5.0 (0.5) years old at baseline (7.0 at follow-up)	Netherlands	Longitudinal-Prospective Cohort	Outdoor play duration Baseline: 10.3 h/wk. Follow-up: 11.4 h/wk
Riiser (2019) [113]	English	426	47.8%	5th grader students	Norway	Cross-sectional	Outdoor play duration Total: 52.7 min/d Sedentary: 13.6 min/d Light PA: 26.0 min/d MVPA: 13.1 min/d Total accelerometer counts/min: 1581.3
Schoeppe (2017) [114]	English	3300	51.2%	11.2 (0.8) years old	Australia	Cross-sectional	Outdoor play frequency 6.0 (2.4) time/wk
Sharp (2018) [115]	English	3373	48.4%	33.5 (17.9) months old	Canada	Cross-sectional	NR
Spurrier (2008) [116]	English	280	50.0%	4.8 (0.2) years old	Australia	Cross-sectional	NR
Stone (2014) [117]	English	856	54.6%	11 (0.6) years old	Canada	Cross-sectional	Outdoor play duration (> 2 h/d) Total: 8% Girls: 6%;Boys: 10%
Tandon (2012) [118]	English	8950	49.0%	4.4 (0.01) years old	USA	Cross-sectional	Outdoor time frequency (at least once/d) 51%
Tappe (2013) [119]	English	724	49.3%	9.1 (1.6) years	USA	Cross-sectional	NR
van Rossem (2012) [120]	English	4688	50.1%	36.7 (1.4) months old	Netherlands	Cross-sectional	Outdoor play duration (> 3 h/d) 8.1%
van Stralen (2012)^b^ [31]	English	600	51.0%	9.8 (0.7) years old	Netherlands	Cross-sectional	Outdoor play frequency 4.1 time/wk
Vandewater (2007) [121]	English	1045	48.0%	0-6 years	USA	Cross-sectional	Outdoor play duration 65.5 min/d
Veitch (2010) [122]	English	187	53.0%	9.1 (0.4) years old	Australia	Cross-sectional	NR
Villarreal-Calderon (2002) [123]	English	559	47.6%	10.8 (1.0) years old	Mexico	Cross-sectional	Outdoor time duration Total: 191 min/d Weekday: 156 min/d Weekend: 226 min/d
Wang (2018) [124]	Chinese	2116	NR	3-6 years	China	Cross-sectional	NR
Weir (2006) [125]	English	204	46.6%	Inner city: 7.4 (1.9) years old Suburban: 6.9 (1.6) years old	USA	Cross-sectional	NR
Wen (2009) [126]	English	1362	52.1%	10-12 years	Australia	Cross-sectional	NR
Wijtzes (2014) [127]	English	4726	49.5%	73 (5.9) months old	Netherlands	Cross-sectional	Outdoor play duration (≥ 1 h/d) 66.3%
Wilkie (2018) [128]	English	462	55.0%	10.9 (0.5) years old	England	Cross-sectional	Outdoor time duration > 1 h/d after school: 52.2% > 2 h/d on weekends: 61.9%
Wiseman (2019) [129]	English	138	48.0%	49.9 (5.4) months old	Australia	Cross-sectional	Outdoor play duration Total: 165.4 min/d Weekdays: 11.9 h/week Weekend days: 7.4 h/week
Xu (2016) [130]	English	667	50.0%	2 years old	Australia	Longitudinal-Prospective cohort	Outdoor play duration 138 min/d at baseline
Xu (2017) [131]	English	497 and 415 mother-child dyads	NR	2-3.5 years	Australia	Cross-sectional	Outdoor play duration (≥ 2 h/d) 68.4%
Yoon (2019) [132]	English	3449	51.7%	Pre-kindergarten to Grade 6	USA	Cross-sectional	Outdoor play duration Weekday Total: 81.6 min/d White: 72.5 min/d; Hispanic: 85.6 min/d
Zahl-Thanem (2019) [133]	English	800	50.2%	6 years at baseline	Turkey	Longitudinal-Prospective cohort	Outdoor time duration 156 min/d
Zhai (2018) [134]	Chinese	403	NR	5-12 years	China	Cross-sectional	Outdoor play duration 4.0 h/wk

^a^Mean age (age range when mean age was not available)

^b^An experimental study but only observational data are extracted

*ECEC* Early Childhood Education and Care; *MVPA* Moderate- to Vigorous-Physical Activity; *NR* Not Reported; *PA* Physical Activity; *OP* Outdoor Play; *OT* Outdoor Time

### Specific outcomes investigated

#### Outdoor play

A total of 85 studies examined potential correlates of outdoor play [31–35, 38–41, 44–47, 49–54, 57, 59–65, 67–75, 77, 79, 80, 82, 83, 85–93, 95–103, 106–114, 116–118, 120, 122, 123, 125–130, 133, 135–138]. Among those, 14 studies measured outdoor physical activity [46, 77, 81–83, 87, 92, 93, 96, 102, 106, 118, 119], which were categorized into outdoor play for the present review. A total of 56 studies used proxy-reported outdoor play [33–35, 40, 41, 47, 50, 52–54, 59–61, 63, 64, 68, 69, 73, 75, 77, 79, 80, 82, 83, 85–87, 89–93, 95–98, 101, 106, 111–113, 116–118, 122, 123, 125–129, 133, 135–138] and 15 studies used self-reported outdoor play [39, 51, 57, 62, 70, 71, 74, 83, 99, 100, 103, 107, 108, 114, 130], of which one observation used both proxy- and self-reported data [102]. Ten studies used accelerometry or pedometry (i.e., device-based measures of physical activity that took place outdoors) [34, 35, 48, 72, 77, 86, 113, 117, 119, 122] and another five studies were based on direct observation [30, 43, 45, 108, 109]. Average duration of outdoor play reported from 26 studies ranged between 60.0 to 165.4 min/d (mean or median) or 2.0 to 10.3 h/week. A total of 11 studies reported the frequency of engagement (e.g., times/wk) and another 13 studies reported a proportion of children engaging in outdoor play for a specific time cut-point (e.g., ≥ 1 h/d).

#### Outdoor time

A total of 22 studies examined potential correlates of outdoor time [37, 42, 48, 55, 56, 58, 76, 78, 81, 84, 104, 105, 115, 121, 124, 131, 132, 134]. The majority of studies used proxy-reported outdoor time (*n* = 12) [36, 37, 43, 56, 66, 76, 81, 105, 115, 131, 132], followed by self-reported (*n* = 5) [75, 87, 101, 123, 128], direct observation (*n* = 2) [52, 83], and device-based measures (*n* = 3) [47, 56, 80]. Average duration of outdoor time reported from 12 studies ranged between 41.7 to 240.0 min/d or 6.2 h/wk. A total of two studies reported the frequency of engagement (e.g., times/week) and four reported a proportion of children meeting a specific time cut-point (e.g., ≥ 1 h/d).

### Identified correlates

#### Outdoor play

Table 2 presents potential correlates of outdoor play examined (“Factors within SEM examined”), statistically significant correlates identified (“Association”), and the direction/strength of evidence (“Consistency of evidence”), classified by different levels of SEM (e.g., “INDIVIDUAL”) and their sub-categories (e.g., “Age”, “Sex/gender”). The overarching summary of evidence were also provided by sub-categories of SEM (“Summary of evidence”). Only statistically significant correlates are highlighted in this section. Out of 35 correlates examined at the individual level, 18 correlates showed positive associations while three correlates showed a negative association with outdoor play. Specifically, being part of a dominant racial/ethnic group (e.g., white/Caucasian in Western countries [38, 57, 67, 68, 74, 83, 85, 93, 123, 128, 130], Chinese ethnicity in China [89]), planning skills [31], and perceived sport competence [31] were positively associated with outdoor play. In addition, child autonomy [111] and independence [126], independent mobility [99, 104], child-initiation [45], overall physical activity [51, 104], regular play [44], outdoor play in the past [31], and tummy time frequency in the past [130] were positively associated with outdoor play. In addition, opposing view (i.e., cons) towards sport participation (e.g., if I participate in sports it will cost me too much time) [31], sedentary time [132], time spent eating lunch [66], Mediterranean diet [92], and having surgency/extraversion and negative affectivity temperament [115] were also positively associated with outdoor play. Having female sex/girl gender [34, 41, 44–47, 49, 62, 63, 66, 71, 73, 97, 100, 120, 122, 133], English being an additional language [66, 126], and strengths and difficulties score (i.e., internalizing problems and pro-social behavior) [34] were negatively associated with outdoor play.

**Table 2:** Correlates of outdoor play

Factors within SEM examined	Association		Consistency of association		Summary of evidence (consistent correlate (“+|+” or “–|–”) is in bold)
	Studies	Direction	Consistency (%)	Direction/ strength	
**INDIVIDUAL (18 identified**^3^**/35 examined**^3^**)**					
**Demographic**					‘ + ’ (1/5): **Race/ethnicity (dominant group)** ‘ − ’ (2/5): Sex/gender (girls), English as an additional language
Age (results from 2 to 12 years only; including grade)	[77]^PA^, [82, 92]*, [63]^PA^*, [119]^PA^, [66]*, [126]*, [50]*, [33, 84]*, [90]*, [111, 134, 137]^a:age 7^	∅	14/23 = 61%	∅	
	[34, 111]^a: age 5^, [112]*	+	3/23 = 13%		
	[61, 96]*, [43]*, [44, 64]*, [79]*	–	6/23 = 26%		
Sex/gender (girls)	[82, 92]*, [63]^PA^*, [66]*, [67]^PA^*, [126]*, [129]*, [50]*, [34, 43]*^a^, [78, 79]*, [89]*, [100]^b:wd^, [110]*, [111, 134, 137]	∅	18/41 = 43%	−?	
	[43, 96]*^a^, [97]	+	3/41 = 7%		
	[35]^PA^, [77]^PA^, [102]*, [106]*, [60, 61, 118, 130]*, [33]*, [40, 43]*^a^, [44, 48]^PA^*, [64]*, [95]*, [99]*, [100]^b:we^, [112, 113]*	–	19/41 = 46%		
Race/ethnicity (dominant)	White/Caucasian (ref): [82] (Hispanic), [119]^PA^*(Hispanic), [63]^PA^* (Black and Hispanic), [66]*(Asian), [61]*(non-Hispanic Black, Hispanic, and Other)	∅	5/17 = 29%	++	
	Chinese (ref): [89] (Malay, Indian, and others)* White/Caucasian (ref): [82] (Black), [92]* (Black, Hispanic, and others), [132] (Hispanic), [66] (Black/Hispanic)*, [67]^PA^*, [129]* (not specified), [34] (Black and minority ethnicities), [71] (British Pakistani girls), [84] (Turkish), [120] (Moroccan, Turkish, and Caribbean vs Dutch), [127] (Surinamese-Hindustani, Dutch Antillean, Cape Verdean, Turkish, Moroccan)	+	12/17 = 71%		
Immigration status (immigrated)	[44]	∅	1/1 = 100%	∅	
English as an additional language	[66]*, [126]	–	2/2 = 100%	–	
**Physical**					0/6
Disability status	[66]*	∅	1/1 = 100%	∅	
Weight status (overweight)	[66]*, [119]^PA^, [117]^b:wd^*, [84]*, [97, 104, 113]^PA^*, [111]	∅	8/11 = 73%	∅∅	
	[117]^b:we^*, [34, 137]	–	3/11 = 27%		
Pubertal status	[104]	∅	1/1 = 100%	∅	
Born premature/birth weight	[66]*, [65] ^PA^, [103]^PA^	∅	3/3 = 100%	∅	
Time in neonatal intensive care unit	[66]*	∅	1/1 = 100%	∅	
Health status (healthy)	[66, 82]*, [69]*	∅	9/13 = 69%	∅∅	
Depression	[96]	∅			
Itchy or watery eyes	[69]*	∅			
Asthma attacks	[69]*	∅			
Cough	[69]*	∅			
Trouble sleeping	[69]*	∅			
Feeling tired or having low energy	[69]*	∅			
Conduct problems	[96]	+	1/13 = 8%		
Body pain or discomfort	[69]*	–	3/13 = 23%		
Repeated upset stomach	[69]*	–			
Frequent swollen glands	[69]*	–			
**Psychological**					‘ + ’ (3/8): Cons of OP, planning skills, perceived sport competence
Self-efficacy	[31]	∅	1/1 = 100%	∅	
Perceived barriers	[31]	∅	1/1 = 100%	∅	
Enjoyment	[31]	∅	1/1 = 100%	∅	
Pros of sport participation	[31]	∅	1/1 = 100%	∅	
Cons of sport participation	[31]	+	1/1 = 100%	+	
Planning skills	[31]	+	1/1 = 100%	+	
Perceived sport competence	[31]	+	1/1 = 100%	+	
Preference for non-active things	[122]	∅	1/1 = 100%	∅	
**Behavioral/temperament**					‘ + ’ (11/16): Child autonomy/independence, independent mobility, child-initiation, **Overall PA**, sedentary time, regular play, OP in the past, min/d spent eating lunch, Mediterranean diet, tummy time frequency, temperament^c^ ‘ − ’ (1/16): Strengths and difficulties
Child autonomy/independence	[61]*, [111]^a: age7^	∅	2/4 = 50%	+?	
	[111, 126]^a: age5^	+	2/4 = 50%		
Independent mobility	[99]*, [104]	+	2/2 = 100%	+	
Child-initiation	[45]	+	1/1 = 100%	+	
Overall PA					
PA levels	[72]	∅	1/4 = 25%	∅	
Structured exercise/sport	[104]*	+	3/4 = 75%	++	
Active travel to/from school	[104]*	+			
Dog walking	66]	+			
Screen time/exposure	[118, 121, 129]*, [34]	∅	4/4 = 100%	∅∅	
Sedentary time (min/week)	[132]	+	1/1 = 100%	+	
Regular play	[44]	+	1/1 = 100%	+	
OP in the past	[137]	∅	1/2 = 50%	+?	
	[31]	+	1/2 = 50%		
Number of days breakfast at home (weekly)	[66]*	∅	1/1 = 100%	∅	
Minutes/day spent eating lunch	[66]*	+	1/1 = 100%	+	
Mediterranean diet	[92]	+	1/1 = 100%	+	
Tummy time within one month of birth	[130]	∅	1/1 = 100%	∅	
Tummy time frequency	[130]	+	1/1 = 100%	+	
Strengths and difficulties				–	
Internalizing problems	[34]	–	2/3 = 67%		
Pro-social behavior	[34]	–			
Externalizing conduct	[34]	+	1/3 = 33%		
Temperament				+?	
Surgency/extraversion	[115]	+	2/4 = 50%		
Negative affectivity	[115]^c:boy^	+			
	[115]^c:girl^	∅	2/4 = 50%		
Effortful control	[115]	∅			
**PARENTAL (33 identified**^3^**/65 examined**^3^**)**					
**Parental sociodemographic**					‘ + ’ (2/18): Parent’s race/ethnicity (Caucasian/white); parent’s nationality (native) ‘ − ’ (4/18): Parent’s immigration status, parental education, mother’s education, mother’s employment, vehicle ownership
Age of mother at birth	[66]*	∅	1/1 = 100%	∅	
Age of primary caregiver/mother	[82, 129]*	∅	2/3 = 66%	∅	
	[98]*	–	1/3 = 33%		
Parent’s gender	[98]*	∅	1/1 = 100%	∅	
Parent’s race/ethnicity	[111]	∅	1/3 = 33%	+	
	White/Caucasian (ref): [118] (Hispanic, Black, and Asian) [50], (Asian or Southeast Asian)*	+	2/3 = 76%		
Parent’s immigration status	Born in Switzerland (ref): [74]^PA^ (immigrated)	–	1/1 = 100%	–	
Parental nationality	[130] (Australian born in Australia)	+	1/1 = 100%	+	
Parental education	[67]^PA^*, [122]*, [61]*; [98]*, [33]^a,c: 4-6 boy/girl^, [74]^PA^, [84]*, [111]	∅	8/15 = 53%	−?	
	[32, 33, 118, 132]^a,c: 7-12 boy/girl^, [37, 38, 100]*	–	7/15 = 47%		
Maternal	[66, 82]*, [50]*, [78, 89]*, [39, 120, 137]	∅	7/13 = 54%	−?	
	[59]*, [44] [92]*, [96]*, [124]*, [127]	–	6/13 = 46%		
Paternal	[96]*, [66]*, [78, 89]*, [97, 127]	∅	6/8 = 75%	∅∅	
	[59]*, [124]*	–	2/8 = 25%		
Parental employment (employed)	[122]*, [60]^b:weekday^, [98]*	∅	3/5 = 60%	∅	
	[126]	+	1/5 = 20%		
	[60]^b:weekend^	–	1/5 = 20%		
Maternal (employed/prestige)	[66]*, [96]*, [120, 127]	∅	4/7 = 57%	−?	
	[63, 82, 118]	–	3/7 = 43%		
Paternal (employed/prestige)	[102]*, [128]	∅	2/4 = 50%	∅	
	[99]	+	1/4 = 25%		
	[138]* (white collar vs unemployed or manual worker, craftsman)	–	1/4 = 25%		
Work shift		∅	1/1 = 100%	∅	
SES/household income (high income)	[82, 96]*, [118, 119]^PA^, [66]*, [50]*, [61]*, [74]^PA^, [89, 97, 134]	∅	11/16 = 69%	∅∅	
	[34, 59]*, [89]*	–	3/16 = 19%		
	[98]*, [127]	+	2/16 = 13%		
Vehicle ownership/number of vehicles	[44]	∅	1/2 = 50%	−?	
	[132]	–	1/2 = 50%		
Number of licenses in household	[60, 132]	∅	2/2 = 100%	∅	
Mother’s financial difficulties	[120]	∅	1/1 = 100%	∅	
Marital status/cohabitation	[66, 82, 118]*, [122]*	∅	4/5 = 80%	∅∅	
	[98]*	+	1/5 = 20%		
**Parental health**					‘ − ’ (1/2): Mother’s depression
Parent’s weight status	[67, 82]^PA^*, [120] (pre-pregnancy)	∅	3/4 = 75%	∅	
	[111]	+	1/4 = 25%		
Maternal depression	[82]	∅	1/3 = 33%	–	
	[62, 68]	–	2/3 = 67%		
**Parenting style/practice**					‘ − ’ (3/9): **Hyper-parenting**, constraint parenting, encouraging sleep
Hyper-parenting				–	
Helicopter	[76]	∅	1/4 = 25%		
Little emperor	[76]	–	3/4 = 75%		
Tiger mom	[76]	–			
Concerted cultivation	[76]	–			
Constraint parenting				–	
Avoidance	[81]*	–	2/2 = 100%		
Defensive	[81]*	–			
Monitoring of child’s PA	[111, 112]	∅	2/2 = 100%	∅	
Rules in the household	[129]*(on indoor/outdoor active play), [32]	∅	2/3 = 67%	∅	
	[111]	+	1/3 = 33%		
Use PA to reward/control child’s behavior	[129]*	∅	1/1 = 100%	∅	
Use screen time to reward/control child’s behavior	[129]*	∅	1/1 = 100%	∅	
Limit/monitor/discourage screen time	[129]*, [98, 130]*	∅	3/4 75%	∅	
	[112]	–	1/4 = 25%		
Limit OP due to weather	[129]*	∅	1/1 = 100%	∅	
Encourages sleep	[98]*	–	1/1 = 100%	–	
**Parental psychological characteristics**					‘ + ’ (3/14): **Parental attitude towards activities**, parents informed about playing with child, importance parents ascribed to OP ‘ − ’ (3/14): Family attitude towards OP, intention to improve OP, concerns towards OP/PA
Parental attitude					
Attitude towards nature	[69]*	+	4/5 = 80%	++	
Attitude towards recreation	[69]*	+			
Attitude towards child’s PA	[112]	+			
Attitude towards walking	[132]	+			
Attitude towards OP	[111]	–	1/5 = 20%		
Family attitude towards OP	[111]^a:age7^	∅	1/2 = 50%	−?	
	[111]^a:age5^	–	1/2 = 50%		
Perception/belief/awareness/intention					
Belief that overweight caused be genetic factors	[84]*	∅	1/1 = 100%	∅	
TPB/awareness about being overweight	[84, 130]*	∅	2/2 = 100%	∅	
Belief of being able to give child activities	[130]	∅	1/1 = 100%	∅	
Intention to improve OP	[111]^a:age7^	∅	1/2 = 50%	−?	
	[111]^a:age5^	–	1/2 = 50%		
Concerns					
Mother’s concern towards OP (fearful)	[82]	∅	1/3 = 33%	–	
	[62]	–	1/2 = 67%		
Parental concerns towards child’s PA	[112]	–			
Perceived responsibility towards child’s PA	[112]	∅	1/1 = 100%	∅	
Pressure towards child’s PA	[112]	∅	1/1 = 100%	∅	
Barriers to child’s activity				∅	
Walking/cycling logistics	[119]^PA^	∅	3/4 = 75%		
Walking/cycling route	[119]^PA^	∅			
Perceived lack of appropriate play areas	[119]^PA^	∅			
Crime activity	[119]^PA^	–	1/4 = 25%		
Knowledge/value/importance					
Informed about playing with child	[130]	+	1/1 = 100%	+	
Knowledge of child development	[130]	∅	1/1 = 100%	∅	
Importance/value of PA	[129]*	∅	1/1 = 100%	∅	
Importance parents ascribed to OP	[32]	+	1/1 = 100%	+	
**Parental behavior/modelling**					‘ + ’ (6/11): Parental outdoor activity, parental frequency of walking, parental frequency of organized sport, **parental frequency of overall PA,** parental modelling, Parental habit strength ‘ − ’ (1/11): Explicit modeling and enjoyment of screen time
Parental outdoor activity				+?	
Maternal	[114]	+	1/2 = 50%		
Paternal	[114]	∅	1/2 = 50%		
Frequency of walking				+	
Maternal	[116]*	+	2/2 = 100%		
Paternal	[116]*	+			
Frequency of organized sport				+	
Maternal	[116]*	+	2/2 = 100%		
Paternal	[116]*	+			
Overall PA				++	
Maternal	[118, 124, 130]*	+	3/4 = 75%		
Parental	[124]*	∅	1/4 = 25%		
Modelling	[111]	∅	1/3 = 33%	+	
	[61, 110]	+	2/3 = 67%		
Explicit modeling and enjoyment of PA	[129]*	∅	1/1 = 100%	∅	
Explicit modeling and enjoyment of screen time	[129]*	–	1/1 = 100%	–	
Smoking during pregnancy	[120]	∅	1/1 = 100%	∅	
Breast feeding	[120]	∅	1/1 = 100%	∅	
Frequency of parents buying low-cost food	[66]*	∅	1/1 = 100%	∅	
Parental habit strength	[111]	+	1/1 = 100%	+	
**Parental support**					‘ + ’ (10/11): Co-participation, encouragement, involvement, providing transportation, family visit to OP spaces, emotional support, informational support, instrumental support, companionship, support for PA
Co-participation	[67]^PA^*, [98]*, [137]	+	3/3 = 100%	+	
Encouragement	[129]*, [111]^a:age5^	∅	2/6 = 33%	+?	
	[111]^a:age7^	–	1/6 = 17%		
	[61, 98, 102]*	+	3/6 = 50%		
Transport	[122]	∅	1/2 = 50%	+?	
	[67]^PA^*	+	1/2 = 50%		
Facilitation	[61]	∅	1/1 = 100%	∅	
Involvement	[61]	+	1/1 = 100%	+	
Family visited OP spaces (playground/park/picnic areas)	[92]	+	1/1 = 100%	+	
Emotional	[110]	+	1/1 = 100%	+	
Informational	[110]	+	1/1 = 100%	+	
Instrumental	[129]* (active play but not for sport), [110]	+	2/2 = 100%	+	
Companionship	[110]	+	1/1 = 100%	+	
Support for PA	[98]*	+	1/1 = 100%	+	
**MICROSYSTEM DIMENSIONS (15 identified**^3^**/33 examined**^3^**)**					
**Proximal social environment**					‘ + ’ (6/12): Sibling modelling, grandmother in household, dog/pet ownership, time spent with mother/father, peer support/ modelling and number of regular playmates, other social support ‘ − ’ (2/12): Number of siblings, using only Spanish at home for non-White Hispanic children
Home					
Number of residents in household	[62, 82]	∅	2/2 = 100%	∅	
Family composition	[97]	∅	6/6 = 100%	∅∅	
Nuclear	[78]	∅			
Stem	[78]	∅			
Single parent	[61, 78, 92]	∅			
Single mother	[120]	∅			
Remarried	[78]	∅			
Number of siblings	[32, 44],	∅	3/8 = 38%	−?	
	[132, 134]	+	2/8 = 25%		
	[66]*, [61, 126]	–	3/8 = 38%		
Older sibling	[82]	∅	1/1 = 100%	∅	
Sibling modeling	[111]	+	1/1 = 100%	+	
Grandmother in household	[132]	+	1/1 = 100%	+	
Using only Spanish at home for Hispanic	[132]	–	1/1 = 100%	–	
Dog/pet ownership	[98, 132]*	+	2/2 = 100%	+	
Time spent alone	[96]	∅	1/1 = 100%	∅	
Time spent with mother	[96]	+	2/2 = 100%	+	
Time spent with father	[96]	+			
Peers					
Has many friends in neighborhood	[122]	∅	3/6 = 50%	+?	
Lots of children play in neighborhood	[122]*	∅			
Time spent with peers unsupervised	[96]	∅			
Number of regular playmates	[118]	+	3/6 = 50%		
Peer support	[110]	+			
Peer modeling	[110]	+			
Other social support				+?	
Time spent with adults other than parents	[96]	∅	2/7 = 29%		
Social modeling	[31]	∅			
Play space-Friend’s/relative’s house	[132]	+	4/7 = 57%		
Support/reinforcement from other adults	[129]*	+			
Social support	[31]	+			
Social capital on obesity and child PA	[112]	+			
Social grouping (adult present)	[45]	–	1/7 = 14%		
**Proximal physical environment (Home)**					‘ + ’ (5/21): Detached house, public housing, living close to friends and family, housing price as a reason for choosing the residence, presence of labor-saving devices, electronics in child’s bedroom ‘ − ’ (1/21): Proximity to work as a reason for choosing the residence
Housing type (detached)	[32] (detached, corner house, flat/apartment); [82] (duplex/townhome)	∅	2/5 = 40%	+?	
	[98, 131]*	+	2/5 = 40%		
	[82] (flat/apartment)	–	1/5 = 20%		
Rental property	[32]	∅	1/1 = 100%	∅	
Public housing	[62, 82]	+	2/2 = 100%	+	
Duration of residency	[82] (since 3 years), [62] (3-5 years), [122]	∅	3/4 = 75%	∅∅	
	[126]	+	1/4 = 25%		
Degree of high/low rise buildings	[32]	∅	1/1 = 100%	∅	
Degree/presence of unoccupied houses	[32, 33]	∅	2/2 = 100%	∅	
Lives in main arterial or busy through road	[122]	∅	1/1 = 100%	∅	
Lives in cul-de-sac	[122]	∅	1/1 = 100%	∅	
Lives close to friends/family					
Close to friends	[34]	∅	1/1 = 100%	∅	
Close to family	[34]	∅	1/1 = 100%	∅	
Close to friends and family	[34]	+	1/1 = 100%	+	
Chose home due to location of kindergarten	[66]*	∅	1/1 = 100%	∅	
Reasons for choosing current residence (housing price)	[132]	+	1/1 = 100%	+	
Reasons for choosing current residence (proximity to work)	[132]	–	1/1 = 100%	–	
Family chose kindergarten	[66]*	∅	1/1 = 100%	∅	
Absence of garden	[32]	∅	1/1 = 100%	∅	
Maintenance of houses	[33]	∅	1/1 = 100%	∅	
Home yard				∅	
Size	[36] (front, back, & total), [122]*	∅	2/3 = 67%		
	[116]*	+	1/3 = 33%		
Natural features and play areas	[36]	∅	5/7 = 71%		
Portable equipment	[36]	∅			
Lawn quality	[36]	∅			
Flowers	[36]	∅			
Herbs and vegetables	[36]	∅			
Fixed equipment	[36]	+	2/7 = 29%		
Number of items of outdoor play equipment	[116]*	+			
Presence of labor-saving devices	[116]*	+	1/1 = 100%	+	
Number of electronic devices in the household	[32]	∅	1/1 = 100%	∅	
Electronic devices in child’s bedroom	[32]	+	1/1 = 100%	+	
**INSTITUTIONAL (12 identified**^3^**/44 examined**^3^**)**					
**Timing**					‘ + ’ (1/3): Scheduling for study
Time of the day	[66]* (hours of before/after school), [134]	∅	2/2 = 100%	∅	
Scheduling for study (partial study > morning/full study > afternoon study)	[38]	+	1/1 = 100%	+	
Days of the week (weekday)	[47]^PA^, [90]*, [134]	∅	3/5 = 60%	∅	
	[64]*	+	1/5 = 20%		
	[86]^PA^*	–	1/5 = 20%		
**Childcare/school environment**					‘ + ’ (5/41): Hours in ECEC, ≥ 50% of educators with level 2/3 certification in ECEC^a^, number of play areas in ECEC^a^, % time on child-centered practices in ECEC, receiving free lunch at school ‘ − ’ (6/41): % small class activities, play space-school, child density, supervising teachers^c^, recess duration, ground surface^c^
ECEC					
Attending daycare/childcare/kindergarten	[62, 66, 82]*, [44, 120]	∅	5/7 = 72%	∅∅	
	[50, 118]	–	2/7 = 28%		
Duration in ECEC care	[66]*	+	1/1 = 100%	+	
Class size	[66, 105]*	∅	1/1 = 100%	∅	
Teacher’s education level (postgraduate or higher)	[66]*	–	1/2 = 50%	?	
	[63]^PA^*	+	1/2 = 50%		
Hours received teacher’s aid	[66]*	∅	1/1 = 100%	∅	
Number of educators (part-time)	[105]	∅	2/2 = 100%	∅	
Number of educators (full-time)	[105]	∅			
≥ 50% of educators with level 2/3 certification	[105]	∅	1/2 = 50%	+?	
	[105]	+	1/2 = 50%		
Accreditation status	[105]	∅	1/1 = 100%	∅	
Centre location (rural vs large)	[105]	∅	1/1 = 100%	∅	
Professional development frequency	[105]	∅	1/1 = 100%	∅	
Professional development topics	[105]	∅	1/1 = 100%	∅	
Family education frequency	[105]	∅	1/1 = 100%	∅	
Family education topics	[105]	∅	1/1 = 100%	∅	
Space to run	[105]	∅	1/1 = 100%	∅	
Play areas	[105]	∅	1/2 = 50%	+?	
	[105]	+	1/2 = 50%		
Portable play equipment	[105]	∅	1/1 = 100%	∅	
Equipment availability	[105]	∅	1/1 = 100%	∅	
Amount of equipment	[105]	∅	1/1 = 100%	∅	
Written policy	[105]	∅	1/1 = 100%	∅	
Policy components	[105]	∅	1/1 = 100%	∅	
% whole class activities	[66]*	∅	1/1 = 100%	∅	
% small class activities	[66]*	–	1/1 = 100%	–	
% time on child-centered practices	[66]*	+	1/1 = 100%	+	
% time on teacher-centered practices	[66]*	∅	1/1 = 100%	∅	
Play group or other education program	[130]	∅	1/1 = 100%	∅	
School					
School type (Public)	[107, 137]	∅	2/2 = 100%	∅	
School-level ethnic density	[39]^PA^	∅	1/1 = 100%	∅	
Free lunch	[77]^PA^	∅	1/1 = 100%	+	
Special lunch program	[132]	+	1/1 = 100%	∅	
Play space-school	[132]	–	1/1 = 100%	–	
Summer camps	[63]^PA^*	∅	1/1 = 100%	∅	
After school programs	[63]^PA^*	∅	1/1 = 100%	∅	
School playground environment during recess					
Child density (number of children/m^2^)	[48]^PA^*	–	1/1 = 100%	–	
Presence of less supervising teachers	[48]^PA^*^c:boy^	∅	1/2 = 50%	−?	
	[48]^PA^*^c:girl^	–	1/2 = 50%		
Recess duration	[48]^PA^*	–	1/1 = 100%	–	
Aiming equipment	[48]^PA^*	∅	1/1 = 100%	∅	
Playing equipment	[48]^PA^*	∅	1/1 = 100%	∅	
Hard ground surface	[48]^PA^*^c:girl^	∅	1/2 = 50%	−?	
	[48]^PA^*^c:boy^	–	1/2 = 50%		
Ground markings	[48]^PA^*	∅	1/1 = 100%	∅	
Vegetation	[48]^PA^*	∅	1/1 = 100%	∅	
Height differences	[48]^PA^*	∅	1/1 = 100%	∅	
Toys	[48]^PA^*	∅	1/1 = 100%	∅	
**MACROSYSTEM DIMENSIONS/COMMUNITY (28 identified**^3^**/98 examined**^3^**)**					
**Built environment**					‘ + ’ (13/70): Number of learning centers, play space, open space, yard, playground, sidewalks, pedestrian amenities, neighborhood greenness, % of segments with low volume roads, traffic calming measure, roundabouts, % distance to kindergarten, distance to nature, aesthetics ‘ − ’ (6/70): walkability score, # of segments with path obstruction, density of crashes, density of intersections, nuisance, neighborhood physical disorder
General environmental characteristics					
General impression	[33]	∅	1/1 = 100%	∅	
Type of neighborhood (city green)	[32] (city green or town center)	∅	1/1 = 100%	∅	
GIS-based PA environment	[119]^PA^	∅	1/1 = 100%	∅	
Physical environment constraint	[104]	∅	1/1 = 100%	∅	
Availability					
Number of formal OP facilities per km^2^	[33]	∅	1/1 = 100%	∅	
Number of learning centers	[66]*	+	1/1 = 100%	+	
Recreation/PA/sport facilities	[63]^PA^*, [88, 134]	∅	3/5 = 60%	+?	
	[119]^PA^, [112]	+	2/5 = 40%		
Play space	[104]	∅	1/3 = 33%	+	
	[38, 132]	+	2/3 = 67%		
Open space	[45]	+	1/1 = 100%	+	
Green space	[32, 33]	∅	2/2 = 100%	∅	
Garden and parks	[63]^PA^*, [60]	∅	2/3 = 67%	∅	
	[44]	–	1/3 = 33%		
Water	[32, 33]	∅	2/3 = 67%	∅	
	[132]	+	1/3 = 33%		
Yard	[92]	+	1/1 = 100%	+	
Playground	[92]	∅	1/3 = 33%	+?	
	[63, 132]^PA^*	+	2/3 = 67%		
Local shops, restaurants, shopping centers, playgrounds, and open spaces	[60]^b:weekday^; [104]	∅	2/3 = 67%	∅	
	[60]^b:weekend^	+	1/3 = 33%		
Travel/Traffic					
Bike lanes	[33]	∅	1/1 = 100%	∅	
Sidewalks	[60]	∅	1/2 = 50%	+?	
	[33]	+	1/2 = 50%		
Walkability	[131]	∅	1/2 = 50%	−?	
	[88]	–	1/2 = 50%		
Pedestrian crossings with traffic lights	[33]^a,c: age 4-6 girls;^ [33]^a,c: 7-9 boys,^; [33]^a,c: 10-12 girls^; [33]^a,c: 10-12 boys^	∅	4/6 = 67%	∅∅	
	[33]^a,c: age 7-9 girls^	–	1/6 = 17%		
	[33]^a,c: age 4-6 boys^	+	1/6 = 17%		
Pedestrian crossings without traffic lights	[33]^a,c: age 4-6 boys^; [33]^a,c: 7-9 girls,^; [33]^a,c: 10-12 girls^; [33]^a,c: 10-12boys^	∅	4/6 = 67%	∅∅	
	[33]^a,c: age 4-6 girls^; [33]^a,c: age 7-9 boys^	+	2/6 = 33%		
Pedestrian amenities	[88]	+	1/1 = 100%	+	
Proportion of segments with path obstruction	[88]	–	1/1 = 100%	–	
Proportion of segments with low volume roads	[88]^c: girls^	∅	1/2 = 50%	+?	
	[88]^c: boys^	+	1/2 = 50%		
Traffic lights	[33]	∅	1/1 = 100%	∅	
Traffic calming	[61]	+	1/1 = 100%	+	
Refuges/safety islands	[33]	∅	1/1 = 100%	∅	
Parallel parking spaces	[33]	∅	1/1 = 100%	∅	
Parking lots	[33]	∅	1/1 = 100%	∅	
Speed bumps	[33]	∅	1/1 = 100%	∅	
Home zones	[33]	∅	1/1 = 100%	∅	
30 km/h zones	[33]	∅	1/1 = 100%	∅	
Roundabouts	[33]	+	1/1 = 100%	+	
Continuity of the road	[134]	∅	1/1 = 100%	∅	
Stop at the side of the road	[134]	∅	1/1 = 100%	∅	
Density of crashes	[132]	–	1/1 = 100%	–	
Density of intersections	[33, 132]	–	2/2 = 100%	–	
Traffic volume and speed	[33, 131, 134]	∅	3/3 = 100%	∅	
Traffic safety	[119]^PA^*, [60]^b:weekend^; [104]^c: boys^, [112, 134]	∅	5/8 = 50%	∅	
	[44, 60]^b:weekday^	–	2/8 = 25%		
	[104]^c: girls^	+	1/8 = 13%		
Traffic accessibility	[134]	∅	1/1 = 100%	∅	
Distance to facilities	[32]	∅	1/1 = 100%	∅	
Distance to kindergarten	[66]*	+	1/1 = 100%	+	
Distance to school	[60, 104]	∅	1/1 = 100%	∅	
Distance to nature	[85]*	+	1/1 = 100%	+	
Distance to park	[60]	∅	1/1 = 100%	∅	
Distance to best friends’ house	[104]	∅	1/1 = 100%	∅	
Distance to stores	[119]^PA^*	∅	1/1 = 100%	∅	
Distance to play areas	[119]^PA^*	∅	1/1 = 100%	∅	
Quality					
OP facilities	[33, 134]	∅	2/2 = 100%	∅	
Green space	[33]	∅	1/1 = 100%	∅	
Neighborhood greenness	[67]^PA^	+	1/1 = 100%	+	
Water	[32, 33]	∅	2/2 = 100%	∅	
Sidewalks	[32, 33, 60]	∅	3/3 = 100%	∅	
Bike lanes	[32, 33]	∅	2/2 = 100%	∅	
Good parks/playgrounds	[122, 131]	∅	2/2 = 100%	∅	
Diversity of routes	[32]	∅	1/1 = 100%	∅	
Satisfaction with play facilities	[32]	∅	1/1 = 100%	∅	
Satisfaction with public green spaces	[32]	∅	1/1 = 100%	∅	
Functionality	[112]	∅	1/1 = 100%	∅	
Aesthetics	[104]	∅	1/2 = 50%	+	
	[119]^PA^	+	1/2 = 50%		
Attractiveness	[111, 112]	∅	2/2 = 100%	∅	
Dog walking area	[33]	∅	1/1 = 100%	∅	
Litter basket for dog waste	[33]	∅	1/1 = 100%	∅	
Trash/litter	[32]	∅	1/1 = 100%	∅	
Dog waste	[32]	∅	1/1 = 100%	∅	
Graffiti	[33]	∅	1/1 = 100%	∅	
Vandalism	[33]	∅	1/1 = 100%	∅	
Dark spaces	[33]	∅	1/1 = 100%	∅	
Nuisance	[104]^c: boys^	∅	1/2 = 50%	−?	
	[104]^c: girls^	–	1/2 = 50%		
Neighborhood physical disorder	[82]	–	1/1 = 100%	–	
**Sociocultural environment**					‘ + ’ (5/17): Social norm, social cohesion, neighbourhood relationships, child friendliness, media message promoting active transport ‘ − ’ (1/17): Social safety
Social norm for PA	[104]	+	1/1 = 100%	+	
Social safety	[32]	∅	1/1 = 100%	∅	
	[60]	–	1/1 = 100%	–	
Social cohesion	[32]	∅	1/2 = 50%	+?	
	[60]	+	1/2 = 50%		
Satisfaction with social contact	[32]	∅	1/1 = 100%	∅	
Neighborhood collective efficacy	[62]	∅	1/1 = 100%	∅	
Neighborhood relationships	[134]	+	1/1 = 100%	+	
Neighborhood SES	[32, 62]	∅	2/2 = 100%	∅	
Neighborhood ethnic density	[39]	∅	1/1 = 100%	∅	
Neighborhood poverty	[82]	∅	1/1 = 100%	∅	
Neighborhood deprivation	[81]*, [60, 104]	∅	3/3 = 100%	∅	
Good neighborhood to bring up child	[122]	∅	1/1 = 100%	∅	
Neighborhood safety	[46, 102, 118, 119]^PA^, [60, 104, 111, 122, 126]	∅	10/11 = 91%	∅	
	[61]	+	1/11 = 9%		
Safety without supervision	[111]	∅	1/1 = 100%	∅	
Safe for outdoor play	[131]	∅	1/1 = 100%	∅	
Neighborhood crime	[122]	∅	1/3 = 33%	∅	
	[61]	+	1/3 = 33%		
	[44]	–	1/3 = 33%		
Child friendliness	[111]	∅	1/2 = 50%	+?	
	[99]*	+	1/2 = 50%		
Media message promoting walking/biking to school	[132]	+	1/1 = 100	+	
**Playground environment**					‘ + ’ (2/11): Play facility provision, feature density^c^ ‘ − ’ (1/11): Naturalness
Physical					
Density	[109]	∅	1/1 = 100%	∅	
Size	[108]	∅	1/1 = 100%	∅	
Aesthetics	[108]	∅	1/1 = 100%	∅	
Cleanliness	[108]	∅	1/1 = 100%	∅	
Play facility quality	[108]	∅	1/1 = 100%	∅	
Division of functional areas	[108]	∅	1/1 = 100%	∅	
Provision of multi-purpose areas	[108]	∅	1/1 = 100%	∅	
Playground improvement made	[30]	∅	1/1 = 100%	∅	
Naturalness	[108]	–	1/1 = 100%	–	
Play facility provision	[108]	+	1/1 = 100%	+	
Feature density	[35]^PA^*^c:boy^	∅	1/2 = 50%	+?	
	[35]^PA^*^c:girl^	+	1/2 = 50%		
Social				∅∅	
Group size	[109]	∅	1/1 = 100%		
Presence of active children	[109]	∅	1/1 = 100%		
Presence of same sex children	[109]	∅	1/1 = 100%		
Presence of opposite sex children	[109]	∅	1/1 = 100%		
Presence of same sex adults	[109]	∅	1/1 = 100%		
Presences of opposite sex adults	[109]	∅	1/1 = 100%		
**PHYSCIAL ECOLOGY/PRESSURE FOR MACROSYSTEM CHANGE (5 identified**^3^**/12 examined**^3^**)**					
Season (Fall/Winter)	[112]*	∅	1/7 = 14%	−−	‘ + ’ (3/12): Temperature, % of high-intensity development, population size of municipality ‘ − ’ (2/12): **Season (Fall/Winter)**, COVID-19
	[105]*, [50, 82]*, [64]*, [86]^PA^*, [37]*	–	6/7 = 86%		
Temperature	[85]*	+	1/1 = 100%	+	
Daylight time	[104]	∅	1/1 = 100%	∅	
Environmental coordination	[134]	∅	1/1 = 100%	∅	
Environmental safety	[134]	∅	1/1 = 100%	∅	
Region (coastal)	[89]*	∅	1/1 = 100%	∅	
Mixed land use	[33, 134]	∅	2/2 = 100%	∅	
% of high-intensity development	[132]	+	1/1 = 100%	+	
Population size of municipality	[131]	∅	1/2 = 50%	+?	
	[61]	+	1/2 = 50%		
Rurality (vs suburban and/or urban)	[125]*, [32, 33, 61] (residential density), [78] (with parents working in large cities), [89]*, [99]*	∅	7/10 = 70%	∅∅	
	[59]*; [136]*; [134]	+	3/10 = 30%		
COVID-19	[98]*	–	1/1 = 100%	–	
Country membership	[49]*	Poland > Denmark > Italy			

^1^Direction: ‘ ∅ ’ = No association; ‘ + ’ = Positive association; ‘ − ’ = Negative association

^2^Consistency: 0-33% observations reporting an association (‘ ∅ ’: No association); 34-59% observations reporting an association (‘ + ?’: Positive association; ‘ − ?’: negative association); 60-100% observations reporting an association (‘ + ’: Positive association; ‘ − ’: Negative association). When 60-100% of ≥ 4 observations reporting an association (‘ ++ ’: Consistent positive association; ‘ −− ’: Consistent negative association; ‘ ∅∅ ’: Consistent no association)

^3^Factors *examined* indicate the correlates that are hypothesized and tested in different studies while factors *identified* indicate the correlates that are potentially important based on the consistency of association

*Unadjusted findings only

^a^Age-stratified findings available

^b^Weekday-weekend stratified findings available

^c^Sex/gender-stratified findings available

^PA^Physical activity during outdoor play was measured

*OP* Outdoor Play; *PA* Physical Activity; *SEM* Socio-ecological Modelling

Out of 65 potential correlates examined at the parental level, 32 correlates showed positive association while 12 correlates showed negative association with outdoor play. Briefly, parent being part of the dominant racial/ethnic group [50, 118], having the dominant nationality [130], parents holding positive attitude towards outdoors/outdoor activities [69, 112, 132], being informed about playing with child [130], ascribing importance to child’s outdoor play [32], parental engagement in different types of physical activities [114, 116, 118, 124, 130] and modelling [61, 110], parental habit strength [111], and parental support [61, 67, 92, 102, 110, 111, 122, 129] were positively associated with outdoor play. On the other hand, having immigrated [74] or higher educated parents [32, 33, 37, 38, 100, 118, 132], having higher educated [44, 59, 92, 96, 124, 127] or working mother [62, 82, 118], number of cars at home [132], having a mother with depression [62, 68], hyper-parenting [76], constraint parenting [81], family holding positive attitude towards outdoor play [111], parent’s intention to improve outdoor play [111], parental concerns towards outdoor play [62] or physical activity [112] were negatively associated with children’s outdoor play. One study examined parental correlates of outdoor play during COVID-19 [135] and found that being encouraged to have adequate sleep was also negatively associated with outdoor play while parental support, particularly co-participation and encouragement, was positively associated with outdoor play among children.

Within microsystem dimensions, out of 33 correlates examined, 11 positive and three negative correlates were identified. Positive correlates of child’s outdoor play within the proximal social environment included sibling modelling [111], peer support and modelling [110], number of regular playmates [118], dog/pet ownership [132, 135], living with grandmother among non-White Hispanic children in the US [132], and time spent with mother/father [96]. Negative correlates included number of siblings [61, 66, 126] and using only Spanish at home for non-White Hispanic children in the US [132]. Within the proximal physical environment, living in a detached home [131, 135] or public housing [62, 82], living close to friends and family [34], choosing the residence based on housing price [132], having labor-saving devices at home [116], and having electronics in the child’s bedroom [32] were positively associated with outdoor play while proximity to work as a reason for choosing the residence [132] was negatively associated with outdoor play among children.

At the institutional level, out of 44 correlates tested, six positive and six negative correlates were found. Specifically, hours in ECEC [66], having more than half of the educators with level 2/3 certification [105], number of play areas [105], % time on child-centered practices [66], scheduling for study time (partial day vs morning/full/afternoon day) [38], and receiving free lunch at school [77] were positively associated with outdoor play. Negative correlates included proportion of small class activities within ECEC [66], school being a major play space versus neighborhood streets or friend’s/relative’s house [132], and child density, recess duration, hard ground surface (for boys only), and presence of less supervising teachers (for girls only) in school playground [48].

At the macrosystem and community level, potential correlates were classified into three major categories: built environment (69 correlates), sociocultural environment (17 correlates), and playground environment (11 correlates). Built environment had four sub-categories: general environmental characteristics, availability, travel/traffic-related, and quality. Out of 70 correlates tested within the built environment 13 positive correlates and six negative correlates were found. Availability of learning centers [66], recreation/PA/sport facilities [112, 119], play space [38, 132], open space [45], yard space [92], and playground areas [92] were positively associated with outdoor play. Also, having sidewalks [33], pedestrian amenities [88], or roundabouts [33], % of segment with low volume roads (boys only) [88], distance to kindergarten [66], and distance to nature [85] were the positive correlates. Walkability [88], % of segments with path obstruction [88], and density of traffic crashes [132] or intersections [33, 132] were negatively associated with outdoor play. As for quality of the built environment, neighborhood greenness [67] and aesthetics [119] were positively, while nuisance (only for girls) [104] and neighborhood physical disorder [82] were negatively, associated with outdoor play. Out of 17 potential correlates included within the sociocultural environment, five positive correlates included social norms [104], social cohesion [60], neighborhood relationships [134], child friendliness [99], and media message promoting active transport [132] and one negative correlate included social safety (‘stranger danger’) [60]. Within the playground environment, play facility provision [108] and feature density [35] were positively associated while naturalness [108] was negatively associated with outdoor play.

In the most distal layer of SEM (physical ecology/pressure for macrosystem change), three positive and two negative correlates out of 12 were found. Specifically, temperature [85], % of high intensity development [132] and population size [61] were positively associated while cold seasons/climate [37, 50, 64, 82, 86, 105] and the current COVID-19 pandemic [135] were negatively associated with outdoor play.

The correlates that were consistently not associated with outdoor play (“ ∅∅ ”) included weight status [66, 84, 97, 104, 111, 113, 117, 119], health status [66, 69, 82, 96], screen time/exposure [34, 118, 121, 129], father’s education [66, 78, 89, 96, 97, 127], SES/household income [60, 67, 73, 75, 83, 90, 99, 102, 119, 121, 137], parental marital status or cohabitation [66, 82, 118, 122], family composition [61, 78, 92, 97, 120], duration of residency in their current neighborhood [62, 82, 122], attendance to ECEC [44, 62, 66, 82, 120], pedestrian crossing with or without traffic lights [33], social aspects of the playground environment (e.g., group size, presence of active children, presence of children and adults by sex) [109], and rurality [32, 33, 61, 78, 89, 99, 125].

#### Outdoor time

Table 3 presents potential correlates of outdoor time examined (“Factors within SEM examined”), statistically significant correlates identified (“Association”), and the direction/strength of evidence (“Consistency of evidence”), classified by different levels of SEM (e.g., “INDIVIDUAL”) and their sub-categories (e.g., “Age”, “Sex/gender”). The overarching summary of evidence were also provided by sub-categories of SEM (“Summary of evidence”). Only statistically significant correlates are highlighted in this section. Of the 10 individual level correlates examined, two positive and five negative correlates were identified. High physical activity levels [55, 133] and having outdoor tendencies [54] were positively, while being a girl [53, 54, 75, 80, 87, 93, 123, 128], African-American in the US [87], immigrant [55], or overweight [101] or having indoor tendencies [54] were negatively, associated with outdoor time. Out of 13 parental level correlates tested, five positive correlates included parental education [98], parental attitude towards nature [69], parental concerns about crime safety (for weekend days only) [128], and parental encouragement (for girls only) [54] and one negative correlate included having no adults to supervise active play outside after school [54].

**Table 3:** Correlates of outdoor time

Factors within SEM examined	Association		Consistency of association		Summary of evidence (consistent correlate (“+ +” or “– –”) is in bold)
	Studies	Direction	Consistency (%)	Direction/ strength	
**INDIVIDUAL (7 identified**^3^**/10 examined**^3^**)**					
**Demographic**					**‘** ***−*** ’ (3/4): Sex/gender (girls), Race/ethnicity (African Americans), immigration status (immigrated)
Age (results from 2 to 12 years only; including grade)	[83]*, [98, 128]^b:weekday^	∅	3/7 = 43%	∅	
	[87]*, [128]^b:weekend^	+	2/7 = 29%		
	[54]*; [55]*	–	2/7 = 29%		
Sex/Gender (girls)	[41]*; [42]*; [55]*; [56]*; [75]*; [83]*; [128]^b:weekend^	∅	7/15 = 47%	−?	
	[53]*; [54]* [75];*; [80]*; [87]*; [93]*; [123]*; [128]^b:weekday^	–	8/15 = 53%		
Race/ethnicity (African American)	[87] (Caucasian/Hispanic)*	–	1/1 = 100%	–	
Immigration status (immigrated)	[55]*	–	1/1 = 100%	–	
**Physical**					**‘** ***−*** ’ (1/2): Weight status (overweight)
Weight status (overweight)	[101]	–	1/1 = 100%	–	
Health status (healthy)	[69]*	∅	5/7 = 71%	∅∅	
Nasal congestion	[69]*	∅			
Asthma attacks	[69]*	∅			
Frequent swollen glands	[69]*	∅			
Diabetes	[69]*	∅			
Trouble sleeping	[69]*	–	2/7 = 29%		
Feeling tired or having low energy	[69]*	–			
**Behavioral/Temperament**					**‘** ***+*** ’ (2/4): Physical activity levels, outdoor tendencies **‘** ***−*** ’ (1/4): Indoor tendencies
Physical activity levels	[55]; [133]* (MVPA)	+	2/2 = 100%	+	
Screen time	[128]^b:weekend^	∅	1/3 = 33%	∅	
	[128]^b:weekday for computer use^	+	1/3 = 33%		
	[128]^b:weekday for tv viewing^	–	1/3 = 33%		
Outdoor tendencies	[54]	+	1/1 = 100%	+	
Indoor tendencies	[54]	–	1/1 = 100%	–	
**PARENTAL (5 identified**^3^**/13 examined**^3^**)**					
**Parental sociodemographic**					**‘** ***+*** ’ (1/5): Parental education
Parental education	[75]* (father’s)	∅	1/2 = 50%	+?	
	[98]	+	1/2 = 50%		
Parent in workforce	[75]*	∅	1/1 = 100%	∅	
SES/Household income	[75]*	∅	1/3 = 33%	∅	
	[128]	+	1/3 = 33%		
	[55]*	–	1/3 = 33%		
Car ownership	[98]	∅	1/1 = 100%	∅	
Home ownership	[98]	∅	1/1 = 100%	∅	
**Parenting style/practice**					**‘** ***−*** ’ (1/3): No adults to supervise active play outside after school
Rules and restrictions	[54]	∅	1/1 = 100%	∅	
Child must be supervised while playing outside	[54]	∅	1/1 = 100%	∅	
No adults to supervise active play outside after school	[54]	–	1/1 = 100%	–	
**Parental psychological characteristics**					
Attitude					**‘** ***+*** ’ (2/4): Parental attitude towards nature, crime safety^b^
Towards nature	[69]*	+	1/1 = 100%	+	
Towards child’s outdoor recreation	[69]*	∅	1/1 = 100%	∅	
Concerns					
Traffic safety	[128]	∅	1/1 = 100%	∅	
Crime safety	[128]^b:weekend^	∅	1/2 = 50%	+?	
	[128]^b:weekday^	+	1/2 = 50%		
**Parental support**					**‘** ***+*** ’ (1/1): Parental encouragement^c^
Parental encouragement	[54]^c:boys^	∅	1/2 = 50%	+?	
	[54]^c:girls^	+	1/2 = 50%		
**MICROSYSTEM DIMENSIONS (6 identified**^3^**/12 examined**^3^**)**					
**Proximal social environment**					**‘** ***+*** ’ (1/5): Social network
Total number of siblings	[54, 75]	∅	2/2 = 100%	∅	
Dog ownership	[54]	∅	1/1 = 100%	∅	
Social trust and cohesion	[73]	∅	1/1 = 100%	∅	
Social opportunities	[54]	∅	1/1 = 100%	∅	
Social network	[73]	+	1/1 = 100%	+	
**Proximal physical environment (Home)**					**‘** ***+*** ’ (3/8): Housing type (detached), residential building characteristics, screen in child’s bedroom^b^ **‘** ***−*** ’ (2/8): Residential building density, access to media^b^
Housing type (detached)	[55]*	+	1/1 = 100%	+	
Duration of residency	[75]*	∅	1/1 = 100%	∅	
Building characteristics					
Number of stories in child’s residence building	[75]*	∅	1/4 = 25%	+?	
Living in a building with outdoor space	[75]*	+	2/4 = 50%		
Lives in neighbourhood with dead-end	[75]	+			
Level of residence floor	[75]	–	1/4 = 25%		
Density-Total building footprint area	[75]	–	2/2 = 100%	–	
Density-Gross building floor area	[75]*	–			
Access to media	[128]^b:weekend^	∅	1/2 = 50%	−?	
	[128]^b:weekday^	–	1/2 = 50%		
Yard size	[54]	∅	1/1 = 100%	∅	
Home PA opportunities	[54]	∅	1/1 = 100%	∅	
Screen in child’s bedroom				+?	
Computer	[128]	∅	3/5 = 60%		
TV	[128]^b:weekend^	∅			
Non hand-held video game player	[128]^b:weekend^	∅			
TV	[128]^b:weekday^	+	2/5 = 40%		
Non hand-held video game player	[128]^b:weekday^	+			
**INSTITUTIONAL (5 identified**^3^**/5 examined**^3^**)**					
**Timing**					**‘** ***+*** ’ (2/5): Time of the day (during school hours), % total vegetation in childcare center **‘** ***−*** ’ (3/5): Days of the week (weekdays), school-level SES^b^, Shade factor in childcare centre
Time of the day (school hours)	[56] (first hour after school)*	+	1/1 = 100%	+	
Days of the week (weekdays)	[47]	∅	1/2 = 50%	−?	
	[55]*	–	1/2 = 50%		
**Childcare/school Environment**					
School-level SES	[98]^b:weekday^	∅	1/2 = 50%	−?	
	[98]^b:weekend^	–	1/2 = 50%		
% total vegetation in childcare	[52]	+	1/1 = 100%	+	
Shade factor in childcare	[52]	–	1/1 = 100%	–	
Country membership of childcare centers	[70]*	Australia > Canada			
**MACROSYSTEM DIMENSIONS/COMMUNITY (8 identified**^3^**/19 examined**^3^**)**					
**Built Environment**					**‘** ***+*** ’ (3/19): Size of the community (small), adjacent space, neighborhood safety **‘** ***−*** ’ (5/19): Residential area, mixed-use building area, under-construction area, street intersection density, gridiron street pattern
Size of the community (small)	[55]*	+	1/1 = 100%	+	
Land-use (total footprint and gross floor areas)					
Residential area	[75]*	–	1/1 = 100%	–	
Commercial area	[75]*	∅	1/1 = 100%	∅	
Institutional building area	[75]*	∅	1/1 = 100%	∅	
Mixed-use building area	[75]*	–	1/1 = 100%	–	
Under-construction area	[75]*	–	1/1 = 100%	–	
Availability/accessibility					
Adjacent space	[75]	+	1/1 = 100%	+	
Local destinations	[54]	∅	1/1 = 100%	∅	
Travel					
Easy to walk	[73]	+	1/1 = 100%	+	
Street width	[75]*	∅	1/1 = 100%	∅	
Traffic volume	[75]*	∅	1/1 = 100%	∅	
Street capacity-street used mainly by pedestrians and non-motorized vehicles	[75]*	∅	1/1 = 100%	∅	
Street capacity-Street used by all types of vehicles, except buses and trucks	[75]*	∅	1/1 = 100%	∅	
Street capacity-Street used by all types of vehicles	[75]*	∅	1/1 = 100%	∅	
Street intersection density	[75]*	–	1/1 = 100%	–	
Unplanned street development	[75]*	∅	1/1 = 100%	∅	
Street pattern-spontaneous street pattern	[75]*	∅	1/1 = 100%	∅	
Street pattern-gridiron street pattern	[75]*	–	1/1 = 100%	–	
Neighbourhood safety	[75]	+	1/1 = 100%	+	
**PHYSCIAL ECOLOGY/PRESSURE FOR MACROSYSTEM CHANGE (2 identified**^3^**/2 examined**^3^**)**					
**Natural Environment**					**‘** ***+*** ’ (1/2): **Rurality** **‘** ***−*** ’ (1/2): Seasonality
Seasonality (Fall/Winter)	[54]	∅	1/4 = 25%	–	
	[55]*; [56]*; [57]*	–	3/4 = 75%		
Rurality (vs suburban/urban)	[98]	∅	1/5 = 20%	++	
	[57]*, [55]*, [58, 80]*	+	4/5 = 80%		

^1^Direction: ‘∅ ’ = No association; ‘ + ’ = Positive association; ‘ − ’ = Negative association

^2^Consistency: 0-33% observations reporting an association (‘ Ø ’: No association); 34-59% observations reporting an association (‘ + ?’: Positive association; ‘ − ?’: negative association); 60-100% observations reporting an association (‘ + ’: Positive association; ‘ − ’: Negative association). When 60-100% of ≥ 4 observations reporting an association (‘ ++ ’: Consistent positive association; ‘ − − ’: Consistent negative association; ‘ØØ’: Consistent no association)

^3^Factors *examined* indicate the correlates that are hypothesized and tested in different studies while factors *identified* indicate the correlates that are potentially important based on the consistency of association

*Unadjusted findings only

^a^Age-stratified findings available

^b^Weekday-weekend stratified findings available

^c^Sex/gender-stratified findings available

*SEM* Socio-ecological modelling

Within the microsystem level, four positive correlates and one negative correlate out of 12 potential correlates were reported. Positive correlates included having a social network [73], living in a detached home [55] or in a building with outdoor space or with dead-end [75], and having a screen in the child’s bedroom (for weekdays only) [128]. Living in a building with high density [75] and having high access to media (weekdays only) were negatively associated with outdoor time among children [128]. At the institutional level, two positive and three negative correlates were identified. Time of the day (during school hours) [56] and % total vegetation in ECEC [52] were positively while weekdays versus weekend days [55], school-level socio-economic status (for weekend only) [98], and shade factor in ECEC [52] were negatively associated with outdoor time. One study examined childcare/preschools in Australia and childcare centers in Canada and average time spent outdoors within centers was greater among Australian centers (143.8 min/d) than Canadian centers (106.8 min/d) [70].

Out of 19 macrosystem dimensions/community level correlates tested, three positive correlates included being part of a small community [55], having adjacent space [75], and living in a walkable neighborhood [73]. Six negative correlates of outdoor time included total residential footprint/gross residential floor area, total mixed-use footprint/gross mixed-use area, total under-construction footprint/gross under-construction area, street intersection density, and having a gridiron street pattern in the neighborhood [75]. Out of two potential correlates tested for the most distal level of SEM, rurality [55, 57, 58, 80] was positively, while seasonality (cold season) [55–57] was negatively, associated with children’s outdoor time.

Varying indicators of health status was not associated with outdoor time [69].

#### Overall key correlates for outdoor play and outdoor time

**Fig. 2:**
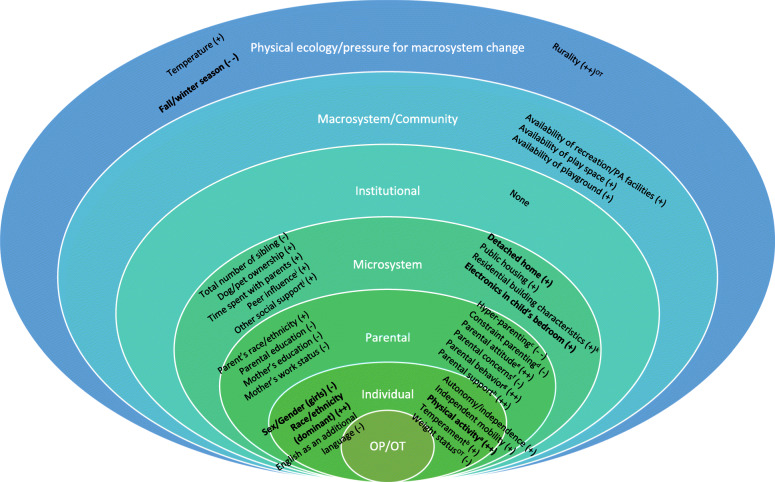
Correlates of outdoor play/time within the socioecological modelling framework. *Note*: Only evidence based on ≥ 2 observations were included in this model. Strong association is indicated in double ‘ ++ ’ or ‘ −− ’. Common correlates of outdoor play and outdoor time are in **bold**. ^OT^ Correlate for outdoor time only; correlate for outdoor play only if not indicated. ^a^ Physical activity included active travel (*n* = 1), structured exercise sport (*n* = 1), regular play (*n* = 1), and dog walking (*n* = 1). ^b^ Temperament included surgency/extraversion (boys and girls) and negative affectivity (boys only). ^c^ Hyper-parenting included little emperor (*n* = 1), tiger mom (*n* = 1) and concerted cultivation (*n* = 1). ^d^ Constraint parenting included avoidance (*n* = 1) and defensive parenting (*n* = 1). ^e^ Parental attitude included attitude towards nature (*n* = 1), attitude toward recreation (*n* = 1), attitude towards child’s physical activity (*n* = 1), and attitude towards walking (*n* = 1). ^f^ Parental concerns included concerns towards child’s outdoor play (*n* = 1) and physical activity (*n* = 1). ^g^ Parental behavior included outdoor activity (*n* = 1), frequency of walking (*n* = 2), frequency of organized sport (*n* = 2), and overall PA (*n* = 3). ^h^ Parental support included co-participation (*n* = 3), encouragement (*n* = 3), proving instrumental support (*n* = 2), and modelling (*n* = 2). ^i^ Peer influence included a number of regular playmates (*n* = 1), peer support (*n* = 1), and peer modeling (*n* = 1). ^j^ Other support included having play space at friend’s or relative’s house (*n* = 1), support/reinforcement from adults other than parents (n – 1), social support (n = 1), and social capital on obesity and child’s physical activity (*n* = 1). ^k^ Residential building characteristics included living in a building with outdoor space (*n* = 1) and living in a neighborhood with dead-end (*n* = 1). IM: Independent mobility; OP: Outdoor play; OT: Outdoor time; PA: Physical activity

Overall key correlates for outdoor play/time are summarized in Fig. 2. In total, 33 correlates were identified as key correlates with seven common correlates across outdoor play/time and five consistent correlates. At the individual level, a total of eight key correlates were identified. Common correlates across outdoor play/time were sex/gender (“ – “for girls) and race/ethnicity (“ ++ ” for dominant racial/ethnic group). Key correlates included child’s autonomy/independence (+), independent mobility (+), physical activity (++), temperament (+), overweight status (–), and English as an additional language (–). Of these, physical activity was identified as a consistent correlate that was positively associated with children’s outdoor play. Ten key correlates were identified at the parental level. Common correlates included parental attitude (++) and parental concerns (+) and consistent correlates included parental attitude (++), parental behavior (++), parental support (++), and hyper parenting (– –). Other key correlates included parent’s race/ethnicity (‘ – ’ for non-dominant racial/ethnic groups), parental education (–), mother’s education (–), mother’s work status (–), and constraint parenting (–). A total of nine key correlates were identified at the microsystem dimensions. Common correlates included living in a detached home (+) and having electronics in the child’s bedroom (+). Other key correlates included total number of siblings (–), dog/pet ownership (+), time spent with parents (+), peer influence (+), other social support (+), living in public housing (+), and residential building characteristics (+). No consistent correlates were found at the microsystem dimensions. No key correlates were identified at the institutional level. At the macrosystem dimensions/community level, three key correlates included availability of recreation/physical activity facilities (+), play space (+), or playground (+). No common or consistent correlates were observed. At the physical ecology/pressure for macrosystem change level, three key correlates were found with two consistent correlates (temperature: ‘ + ’; fall/winter season: ‘ – – ’; rurality: ‘ ++ ’) of which seasonality was also a common correlate for outdoor play/time.

## Discussion

This systematic review used the SEM framework [23, 24] to examine potential correlates of outdoor play/time in children aged 3-12 years. In the 107 studies identified, a total of 287 potential correlates were examined for outdoor play and a total of 61 potential correlates were examined for outdoor time. Of these, 111 correlates for outdoor play and 33 correlates for outdoor time were considered as important. Finally, a total of 33 correlates were identified as key correlates of outdoor play and/or outdoor time, including eight correlates at the individual level, 10 correlates at the parental level, nine in the microsystem dimensions, three at the macrosystem dimension/community level, and three in the physical ecology/pressure for macrosystem change dimension.

Several demographic correlates were examined and identified in this review. In particular, female sex/girl gender and non-dominant racial/ethnic group membership (for both children and parents’) were commonly associated with lower levels of outdoor play/time. Sex/gender and race/ethnicity have been consistently identified as major correlates of other health-related behaviors, such as physical activity and sedentary behavior [139, 140], in this age group. However, because they are not modifiable factors, it is difficult to develop strategies other than targeted interventions for specific population groups. This may explain the limited evidence for long-term effectiveness of targeted interventions based on sociodemographic factors [141]. To better identify correlates of outdoor play/time, taking a more holistic approach towards identifying influencing factors and examining interactions and processes between two or more variables at different levels of SEM may be beneficial. For instance, explaining how sociocultural attitudes and norms interact with sociodemographic factors and, together, influence outdoor play/time may provide more insight into developing tangible solutions to population groups with low levels of social participation in outdoor settings. This review could not identify variables in the meso- or exo-system dimensions due to lack of evidence examining interactions and processes of two or more variables. Future work should therefore explore ‘how’ and ‘why’ children’s or parents’ identity characteristics interact with other variables at proximal and distal physical and social environments (e.g., household income, residence type, peer/social support, neighborhood characteristics). This will allow researchers to elaborate on key mechanisms (i.e., mediators and moderators) that serve as indirect influencing factors for outdoor play/time. The effort to enhance our understanding of the mechanisms can also be done or be paired with qualitative investigation to obtain a thick description [142] of complex sociocultural conditions around the outdoor culture.

Children’s outdoor play/time appears to be influenced by the factors that are proximal to children within SEM. Four out of six consistent correlates (strong evidence) were found in individual and parental levels and the other two were found in the most distal level of the SEM framework. In addition to children’s own physical activity levels being correlated with outdoor play [51, 104]/time [55, 133] at varying degrees, parents seem to play an important role in providing children with outdoor opportunities. Specifically, parents holding positive attitude towards overall physical activity [112, 132] and recreation/nature [69], parents being physically active role models [61, 110, 114, 116, 118, 124, 130], and parents providing support [61, 67, 92, 102, 110, 129, 135, 137] were found to be important, particularly for outdoor play. Parental influence being a strong predictor of outdoor play/time, and physical activity more broadly, has been highlighted in recent work [20, 143]. Important parental correlates of children’s outdoor play in the review done by Boxberger and Reimer [20] were focused on parents’ sociodemographic characteristics (i.e., mother’s ethnicity, mother’s employment status, parents’ education level) as well as one correlate on parental attitude (i.e., importance parents put on outdoor play) and another within the macrosystem/community level (i.e., perceived social cohesion in neighborhood). By having more inclusive criteria of investigation, the results of our systematic review was similar to the correlates of 24-h movement behaviors, which included parental support, modelling, knowledge/belief as well as parents’ sociodemographic factors [143]. Nonetheless, there are gaps in the literature with regards to the influence of family systems on children’s outdoor play/time. Specifically, similar to the individual level correlates, parental level correlates may likely interact within the overarching family systems. For instance, the sociocultural environment of which parents are being part of based on the sociodemographic background of parents and their children may likely influence their practices and support in child-rearing. For example, findings based on qualitative evidence on independent active free play suggested that parental concerns around safety is the main barrier, moderated by child’s age and gender as well as broader societal issues (e.g., reduced sense of community, changes in employment patterns and long work hours) [144]. This further highlights the importance of examining interactions and processes between factors within and across different levels of SEM.

In addition to the role of parents, variables that are most distal were also found to consistently predict children’s outdoor play/time. Specifically, fall/winter season was identified as a consistent, negative correlate for both outdoor play [37, 50, 64, 82, 86, 105] and outdoor time [55–57]. Seasonality is known as an important correlate of children’s overall physical activity [145, 146]. Given that outdoor play/time occurs in outdoors, the role of physical ecology such as weather may be even more critical in affording children opportunity to spend time outdoors. A positive relationship between ambient temperature and outdoor play found in our review also adds to the importance of seasonality. Rurality [55, 57, 58, 80] was also identified as a consistent correlate of outdoor time in our review. Both built and natural environments are important for overall physical activity [145–147]. Although the urban environment is known to be more conducive to certain domains of physical activity such as active transport [147]; our review suggests that the rural environment could be more critical for children spending more time outdoors than urban or suburban environments. In a recent study among Canadian school-aged children living in urban areas, living in a neighborhood with more trees was independently associated with more free-time physical activity [148]. Given the continuing urbanization and development globally, it may be important to conserve natural environments and create more green areas in urban centers.

Lack of studies examining mechanisms (i.e., interactions and processes between different variables) may also explain limited consistency and evidence observed at the higher-level variables such as institutional (*n* = 0), macrosystem/community (*n* = 3), and physical ecology/pressure for macrosystem change (n = 3) levels. In particular, the most frequently studied correlates were macrosystem dimensions/community level correlates for both outdoor play (*n* = 97) and outdoor time (*n* = 19); however, only three variables were identified as key correlates (i.e., availability of recreation/PA facilities, play space, and playgrounds). These correlates may largely depend on neighborhood deprivation or poverty which, in turn, also may reflect household income or type of residence (e.g., social housing), and parental variables (e.g., parental support) or identity variables (e.g., racialized/ethnic minority demographics) that are associated with these characteristics. Another potential reason for the paucity of literature on institutional level correlates included in this review could be due to the eligibility criteria of this review. Specifically, we did not include articles that have examined physical activity at different intensities; therefore, school- or childcare centre-based research examining outdoor physical activity would have been excluded during the screening process. Furthermore, though not captured in this review, consequences of climate change (e.g., increasing frequency of extreme weather events, natural disasters, and air pollution) may likely interact with variables in different levels of SEM to influence children’s outdoor play/time [149, 150].

Additional gaps that are noteworthy to mention are the confusion that exists in the terminology of outdoor play/time, absence of measures of outdoor play/time with established psychometric properties, and heterogeneity of measuring and operationalizing correlates, particularly at the microsystem, institutional, and macrosystem/community levels. Confusion in terminology of outdoor play/time is well-noted in previous literature [20, 151]. In our review, outdoor physical activity [35, 39, 47, 63, 65, 67, 77, 86, 103, 113, 119, 152], outdoor activity, outdoor playtime [38, 68, 100, 137], playground usage [30, 108], active free play [122], outdoor active play [61] and in different settings (e.g., playground, on street, during recess) were observed in addition to outdoor play/time. Establishing clear definitions of outdoor play, outdoor time, and other relevant terms may not only reduce the confusion that exist in the field but may also advance the measurement of outdoor play and outdoor time. In one study, a major discrepancy existed between parent- and child-reported outdoor play. Specifically, among 748 parent-child dyads, 82% of parents reported that their child play more than 30 min/d outdoors while only 3% of their children reported that they play outside more than 30 min/d [102]. Furthermore, the correlates examined were largely heterogeneous, which made it challenging to group different correlates to draw high-level conclusions. For instance, traffic safety may encompass traffic calming (e.g., traffic lights, roundabouts, traffic bumps), volume of motorized vehicle traffic, and the presence of pedestrian infrastructure (e.g., sidewalks, bicycle lanes); however, these variables were considered as individual correlates, rather than being grouped together. The absence of consistent evidence at the institutional and macrosystem/community levels requires future research. Nonetheless, the findings of our review expand and extend on the previous reviews that have examined correlates of outdoor play [20, 21] and offer key correlates that could be important for future intervention programs to promote outdoor play/time among children.

Important considerations should be given in investigating the correlates of outdoor play/time and developing intervention strategies in future research. Specifically, it is important to acknowledge and consider different contexts and conditions in which children are born, live, and play [153]. Giles and colleagues [153] also suggested that benefits and risks for outdoor play may vary across different population groups; therefore, more nuanced investigations, recommendations, and intervention strategies may be required, particularly for children who are underprivileged. In another study [154] exploring how practitioners conceptualize and operationalize nature play, it was suggested that emphasizing measurable outcomes of nature play (e.g., reducing childhood obesity, improving physical literacy, learning about environmental awareness and stewardship) may, in fact, act as a disabling factor in providing more outdoor opportunities in natural settings where children can truly be spontaneous and creative rather than having to experience play defined by adults with measurable goals in mind.

This systematic review provides comprehensive evidence synthesis on the correlates of outdoor play/time, separately and together. The key correlates were also synthesized in great detail based on the strength and direction of evidence as well as the correlates that are common across outdoor play/time or specific to outdoor play or outdoor time. Nevertheless, this study has some notable limitations. The evidence was partially based on unadjusted findings as adjusted findings were often not available. Unadjusted findings were more common at the proximal levels of SEM. For instance, 45 and 88% of evidence that drove sex/gender being a correlate for outdoor play and outdoor time, respectively, were based on unadjusted findings. In addition to English written articles, articles in Chinese, Korean, Spanish, and Portuguese were also searched and included in the review in an effort to be more inclusive of languages other than English. However, 88.0% of the included studies were in English with 82.4% of those coming from Western countries (i.e., West-Europe, North America, Australia, and New Zealand). Also, a total of 14,202 independent articles were screened; however, it is possible that some relevant articles were missed or overlooked. Though we further divided results by age-, sex/gender-, or weekday/weekend sub-categories when the results were inconsistent across the categories of these variables, sub-group analyses were not conducted given that most studies provided overall findings only. Finally, settings, where outdoor play or time occur (e.g., school ground, childcare, playground), may play an important role in further contexualizing the important correlates of outdoor play or time; however, we did not have sufficient number of articles per setting that could lead to making meaningful conclusions.

In addition, due to the heterogeneity across studies included, meta-analysis was not appropriate. Finally, the classification on the consistency of the association of each correlate investigated and potential correlates was made based on previous literature [20, 28, 29], which is not as robust as meta-analyses.

## Conclusions

This systematic review summarized the correlates of outdoor play and outdoor time, separately and together, using the SEM framework. Among children aged 3-12 years, correlates that appear to be important for both outdoor play and outdoor time included boy gender, memberships with the dominant race/ethnic group, being physically active, living in a detached home, having electronics in the child’s bedroom, and warm seasons. For outdoor play only, parental attitude, parental behavior, and parental support, parenting practice may serve as important avenues for future intervention efforts. That being said, in order to promote outdoor play/time where children can be spontaneous and creative, focusing more on children’s play itself as freely-chosen and self-directed while focusing less on adult-led activities and linking outdoor play/time with measurable outcomes (e.g., skills development, reducing obesity) may be important. Rurality appears to be important for outdoor time while the built and social environments may be more critical for outdoor play. Future work should investigate the interactions and processes of more than two variables at the same or different levels of SEM to better understand the interplay of correlates and, thus, to better support outdoor play/time opportunities for children. In investigating correlates and developing intervention strategies, it is important to note that benefits and risks of outdoor play/time may vary across different cultures, countries, and population groups; therefore, special attention should be given to different contexts and conditions in which children are born, live, and play.

## Supplementary Information

**Additional file 1.**

## Data Availability

Not applicable.

## Abbreviations

ECEC: Early Childhood Education and Care
MVPA: Moderate- to vigorous-intensity physical activity
OP: Outdoor play
OT: Outdoor time
PA: Physical activity
SEM: Socio-ecological modelling
