# Supplementary material for: Systematic review of the correlates of outdoor play and time among children aged 3-12 years
Source: Int J Behav Nutr Phys Act. 2021 Mar 18;18:41. doi: 10.1186/s12966-021-01097-9 (PMC7972019; doi:10.1186/s12966-021-01097-9)
Supplement: Supplementary file 1 — Additional file 1. [file 12966_2021_1097_MOESM1_ESM.doc]

Supplementary Table 1. Search strategy

| **Language** | **Key terms** | **Examples of search terms** |
| --- | --- | --- |
| English | **Outdoor:** "outdoor"[All Fields] OR "outdoors"[All Fields]  **Outside:** "outside"[All Fields] OR "outsides"[All Fields]  **Play:** "play and playthings"[MeSH Terms] OR ("play"[All Fields] AND "playthings"[All Fields]) OR "play and playthings"[All Fields] OR "play"[All Fields]  **Time:** "time"[MeSH Terms] OR "time"[All Fields]  **Children:** "child"[MeSH Terms] OR "child"[All Fields] OR "children"[All Fields] OR "child's"[All Fields] OR "children's"[All Fields] OR "childrens"[All Fields] OR "childs"[All Fields] OR “early years” [All Fields]  **Youth:** "adolescent"[MeSH Terms] OR "adolescent"[All Fields] OR "youth"[All Fields] OR "youths"[All Fields] OR "youth's"[All Fields] | MEDLINE Search:  ((outdoor) OR (outside)) AND ((play) OR (time) OR (playtime)) AND ((child*) OR (preschool) OR (youth)) AND (english[Filter]) Filters: English  (((("outdoor"[All Fields] OR "outdoors"[All Fields]) OR ("outside"[All Fields] OR "outsides"[All Fields])) AND ((((("play and playthings"[MeSH Terms] OR ("play"[All Fields] AND "playthings"[All Fields])) OR "play and playthings"[All Fields]) OR "play"[All Fields]) OR ("time"[MeSH Terms] OR "time"[All Fields])) OR "playtime"[All Fields])) AND (("child*"[All Fields] OR "preschool"[All Fields]) OR (((("adolescent"[MeSH Terms] OR "adolescent"[All Fields]) OR "youth"[All Fields]) OR "youths"[All Fields]) OR "youth s"[All Fields]))) AND "english"[Language] |
| Korean | **Outdoor or outside**: 야외, 실외  **Play**: 놀이, 활동, 운동  **Time**: 시간  **Children aged 3-12 years**: 영유아, 아동, 유치원생, 초등학생 | | "야외놀이" or "실외놀이" or "야외활동" and “영유아”; KCI 등재/후보, AHCI | | --- | | |  | | --- | | "실외활동" or "야외운동" or "실외운동" and child*; KCI 등재/후보, AHCI  "외출" and “영유아”; KCI 등재/후보, AHCI | |  | | |
| Mandarin Chinese | **Outdoor time/play:**  户外*('体力活动'+'活动'+'运动'+'体育')  **Children aged 3-12 years:** '幼儿'+'儿童' | (TI=(户外*('体力活动'+'活动'+'运动'+'体育'))*('年龄'+'性别'+'收入'+'经济'+'种族'+'民族'+'移民'+'家庭'+'父母'+'母亲'+'父亲'+'长辈'+'同伴'+'同龄'+'同学'+'邻里'+'邻居'+'组织'+'机构'+'学校'+'幼儿园'+'托儿所'+'托幼'+'社区'+'街道'+'环境'+'建成环境'+'场地'+'建筑布局'+'设施可利用性'+'接近性'+'可及性'+'可达性'+'健身场所'+'设计'+'规划'+'布置'+'活动空间'+'空间特征'+'气候'+'居住密度'+'交通'+'步行指数'+'设施'+'关联'+'政策'+'法律'+'法规')*('幼儿'+'儿童')) OR (KY=(户外*('体力活动'+'活动'+'运动'+'体育'))*('年龄'+'性别'+'收入'+'经济'+'种族'+'民族'+'移民'+'家庭'+'父母'+'母亲'+'父亲'+'长辈'+'同伴'+'同龄'+'同学'+'邻里'+'邻居'+'组织'+'机构'+'学校'+'幼儿园'+'托儿所'+'托幼'+'社区'+'街道'+'环境'+'建成环境'+'场地'+'建筑布局'+'设施可利用性'+'接近性'+'可及性'+'可达性'+'健身场所'+'设计'+'规划'+'布置'+'活动空间'+'空间特征'+'气候'+'居住密度'+'交通'+'步行指数'+'设施'+'关联'+'政策'+'法律'+'法规')*('幼儿'+'儿童')) OR (AB=(户外*('体力活动'+'活动'+'运动'+'体育'))*('年龄'+'性别'+'收入'+'经济'+'种族'+'民族'+'移民'+'家庭'+'父母'+'母亲'+'父亲'+'长辈'+'同伴'+'同龄'+'同学'+'邻里'+'邻居'+'组织'+'机构'+'学校'+'幼儿园'+'托儿所'+'托幼'+'社区'+'街道'+'环境'+'建成环境'+'场地'+'建筑布局'+'设施可利用性'+'接近性'+'可及性'+'可达性'+'健身场所'+'设计'+'规划'+'布置'+'活动空间'+'空间特征'+'气候'+'居住密度'+'交通'+'步行指数'+'设施'+'关联'+'政策'+'法律'+'法规')*('幼儿'+'儿童')) |
| Portuguese | **Outdoor:** "outdoor"[All Fields] OR "outdoors"[All Fields]  **Outside:** "outside"[All Fields] OR "outsides"[All Fields]  **Play:** "play and playthings"[MeSH Terms] OR ("play"[All Fields] AND "playthings"[All Fields]) OR "play and playthings"[All Fields] OR "play"[All Fields]  **Time:** "time"[MeSH Terms] OR "time"[All Fields]  **Children:** "child"[MeSH Terms] OR "child"[All Fields] OR "children"[All Fields] OR "child's"[All Fields] OR "children's"[All Fields] OR "childrens"[All Fields] OR "childs"[All Fields] OR “early years” [All Fields]  **Youth:** "adolescent"[MeSH Terms] OR "adolescent"[All Fields] OR "youth"[All Fields] OR "youths"[All Fields] OR "youth's"[All Fields] | LILACS: ((outdoor) OR (outside)) AND ((play) OR (time) OR (playtime)) AND ((child*) OR (preschool) OR (youth)) Filtered by: LANGUAGES: (Portuguese) |
| Spanish | **Outdoor:** "outdoor"[All Fields] OR "outdoors"[All Fields]  **Outside:** "outside"[All Fields] OR "outsides"[All Fields]  **Play:** "play and playthings"[MeSH Terms] OR ("play"[All Fields] AND "playthings"[All Fields]) OR "play and playthings"[All Fields] OR "play"[All Fields]  **Time:** "time"[MeSH Terms] OR "time"[All Fields]  **Children:** "child"[MeSH Terms] OR "child"[All Fields] OR "children"[All Fields] OR "child's"[All Fields] OR "children's"[All Fields] OR "childrens"[All Fields] OR "childs"[All Fields] OR “early years” [All Fields]  **Youth:** "adolescent"[MeSH Terms] OR "adolescent"[All Fields] OR "youth"[All Fields] OR "youths"[All Fields] OR "youth's"[All Fields] | LATINDEX (via DIALNET): ((outdoor) OR (outside)) AND ((play) OR (time) OR (playtime)) AND ((child*) OR (preschool) OR (youth)) Refined by: LANGUAGES: (SPANISH) Databases= Latindex Timespan=All years |

Supplementary Table 2. Database search results and study selection

| **Language** | | **Databases** | **Date of search** | **Studies identified** | **Duplicates** | **After deduplication** | **Studies Included after title/abstract screening** | **Studies Included after full-text screening** | **% of agreement or Cohen’s** 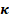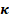 |
| --- | --- | --- | --- | --- | --- | --- | --- | --- | --- |
| English | Initial | MEDLINE | Oct 28, 2019 | 234 |  |  |  |  | Level 1:  90.1%  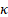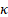 = 0.46  Level 2:  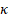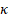 = 0.41 |
|  |  | CINAHL | Oct 29, 2019 | 1341 |  |  |  |  |  |
|  |  | PsychINFO | Oct 28, 2019 | 4079 |  |  |  |  |  |
|  |  | SM&E Index | Oct 29, 2019 | 1024 |  |  |  |  |  |
|  |  | SportDiscus | Oct 29, 2019 | 1090 |  |  |  |  |  |
|  |  | WoS | Nov 4, 2019 | 4529 |  |  |  |  |  |
|  |  | Sub-total |  | 12297 | 1624 | 10673 | 443 | 88 |  |
|  | Top up | MEDLINE | July 27, 2020 | 129 |  |  |  |  |  |
|  |  | CINAHL | July 27, 2020 | 24 |  |  |  |  |  |
|  |  | PsychINFO | July 27, 2020 | 352 |  |  |  |  |  |
|  |  | SM&E Index | July 27, 2020 | 399 |  |  |  |  |  |
|  |  | SportDiscus | July 27, 2020 | 227 |  |  |  |  |  |
|  |  | WoS | July 27, 2020 | 433 |  |  |  |  |  |
|  |  | Sub-total |  | 1319 | 116 | 1203 | 39 | 6 |  |
|  | Total | |  | 13616 | 1740 | 11876 | 482 | **94** |  |
| Korean | | KISS | June 23, 2020 | 238 | 0 | 238 | 13 | **0** | Level 1:  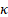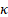 = 0.94  Level 2:  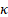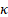 = 0.70 |
| Mandarin Chinese | | [CNKI](http://new.gb.oversea.cnki.net/index/) |  | 1677 |  |  |  |  | Level 1: 97.6%  Level 2: 97.1% |
|  |  | [Wanfang](http://www.wanfangdata.com.cn/index.html?index=true) |  | 366 |  |  |  |  |  |
|  |  | Total |  | 2043 | 280 | 1737 | 35 | **7** |  |
| Portuguese | | SciELO |  | 34 |  |  |  |  | 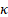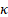 = 0.84 |
|  |  | LILACS |  | 49 |  |  |  |  |  |
|  |  | Studies forwarded |  | 3 |  |  |  |  |  |
|  |  | Total |  | 86 | 28 | 58 | 48 | **3** |  |
| Spanish | | MEDLINE (Spanish) |  | 79 |  |  |  |  | 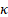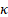 = 0.57 |
|  |  | SciELO |  | 67 |  |  |  |  |  |
|  |  | WoS |  | 32 |  |  |  |  |  |
|  |  | LILACS |  | 37 |  |  |  |  |  |
|  |  | LATINDEX |  | 110 |  |  |  |  |  |
|  |  | Studies forwarded |  | 4 |  |  |  |  |  |
|  |  | Total |  | 329 | 62 | 293 | 56 | **2** |  |
| Hand search for English articles | |  | Nov 5, 2020 |  |  |  |  | 1 |  |
| **Overall** | |  |  | **16312** | **2110** | **14202** | **634** | **107** |  |

Supplementary Table 3. Risk of Bias assessment for observational studies (N = 107)

| **Last name of the First author (year)** | **Selection bias** | **Performance bias** | **Detection bias** | **Attrition bias** | **Selective reporting** | **Other sources of bias** | **Quality rating score** |
| --- | --- | --- | --- | --- | --- | --- | --- |
| Aarts (2010) | High | Unclear | Unclear | Low | Low | Low | 2 |
| Aarts (2012) | Low | Unclear | High | High | Low | Low | 2 |
| Aggio (2017) | Low | Low | Unclear | High | Low | Low | 4 |
| Anthamatten (2014) | Low | Low | Low | Unclear | Low | High | 4 |
| Armstrong (2019) | Low | Unclear | Unclear | High | Low | Low | 3 |
| Bagordo (2017) | Low | Unclear | Unclear | High | Low | Low | 3 |
| Barros (2012) | Low | Low | Low | Low | Low | Low | 6 |
| Barros (2019) | Low | Low | Low | High | Low | Low | 5 |
| Berger (2019) | Low | Low | Low | Low | Low | Low | 6 |
| Berglind (2017) | Low | Unclear | Unclear | Low | Low | Low | 4 |
| Bohn-Goldbaum (2013) | High | High | Low | Low | Low | High | 3 |
| Boldemann (2006) | Low | Low | Unclear | Low | High | High | 3 |
| Boldemann (2011) | Low | Low | Unclear | High | High | High | 2 |
| Bourke (2014) | Low | High | Low | Low | Low | High | 4 |
| Bringhold-Isler (2010) | Low | Unclear | Unclear | Low | Low | Low | 4 |
| Brown (2009) | Low | Low | Low | Low | Unclear | High | 4 |
| Burdette (2005) | Low | Unclear | Unclear | Low | Low | Low | 4 |
| Burgi (2016) | High | Low | Low | Low | Low | Low | 5 |
| Cardon (2008) | Low | Unclear | Low | Low | Low | High | 4 |
| Caroli (2011) | High | Low | High | Unclear | Low | High | 2 |
| Carsley (2017) | Unclear | Unclear | Unclear | Unclear | Low | Low | 2 |
| Christian (2014) | High | Low | Low | Low | Low | Low | 5 |
| Christian (2019) | Unclear | Low | High | Low | Low | High | 3 |
| Cleland (2008) | Low | Low | Low | Low | Low | High | 5 |
| Cleland (2010) | Low | Low | Low | High | Low | Low | 5 |
| Conrad (2013) | Low | Low | Unclear | Low | Low | High | 4 |
| Cooper (2010) | Low | Unclear | Low | Low | High | Unclear | 3 |
| Damore (2002) | Low | High | High | Unclear | Low | High | 2 |
| Donatiello (2013) | Low | Low | Low | Low | Low | Low | 6 |
| Dregval (2009) | Low | Unclear | Unclear | Low | High | Unclear | 2 |
| Faulkner (2015) | Low | Low | Low | Unclear | Low | Low | 5 |
| Ferrao (2015) | Unclear | High | High | Low | Low | Low | 3 |
| Frech (2011) | High | Unclear | Unclear | Low | Low | Low | 3 |
| Galvez (2012) | Unclear | Low | High | Low | Low | Low | 4 |
| Gao (2017) | Unclear | Low | Low | Low | Low | High | 4 |
| Gopinath (2013) | Low | Low | High | High | Low | Low | 4 |
| Gottfried (2017) | Low | Low | Unclear | Low | Low | Low | 5 |
| Grigsby-Toussaint (2011) | Low | Low | Low | Low | Low | Low | 6 |
| Gross (2013) | Low | Low | Low | Low | Low | Low | 6 |
| Hammond (2011) | High | High | High | Low | Low | High | 2 |
| Hinkley (2014) | Low | Low | High | High | Low | High | 3 |
| Hornby-Turner (2014) | Unclear | Low | Low | Low | Low | Low | 5 |
| Howie (2013) | Unclear | Low | Low | Unclear | Low | Unclear | 3 |
| Hunter (2020) | Unclear | Low | High | Low | Low | Low | 4 |
| Imhof (2016) | Low | Unclear | High | Low | Low | Low | 4 |
| Islam (2016) | Low | Unclear | Unclear | Low | Low | Low | 4 |
| Janssen (2015) | Unclear | Low | Low | Low | Low | Low | 5 |
| Jerrett (2013) | Low | Unclear | Low | High | Low | High | 3 |
| Jin MH (2020) | Low | Low | Unclear | Low | Low | Low | 5 |
| Jin F (2020) | High | Low | High | Low | Low | Low | 4 |
| Jones (2009) | High | Low | Low | High | High | High | 2 |
| Kepper (2020) | Low | Low | Low | Low | Low | Low | 6 |
| Kimbro (2011) | Low | Unclear | Unclear | Low | Low | Low | 4 |
| Kneeshaw-Price (2013) | Low | Low | Low | Low | Low | High | 5 |
| Kocken (2012) | Low | Unclear | Low | High | Low | High | 3 |
| Kos (2013) | Unclear | High | High | Unclear | Low | High | 1 |
| Lachowycz (2012) | Low | Low | Low | High | Low | High | 4 |
| Larson (2019) | Low | Low | Low | Low | Low | High | 5 |
| Lee RE (2016) | Low | Low | Unclear | Low | Low | High | 4 |
| Lee ST (2016) | High | Low | Unclear | Unclear | Low | Low | 3 |
| Liu MY (2020) | High | Low | High | Low | Low | Low | 4 |
| Liu TT (2020) | High | Low | High | Low | Low | Low | 4 |
| Marino (2012) | Low | High | High | Low | High | High | 2 |
| Martin (2008) | High | Low | High | Low | High | High | 2 |
| Martino (2019) | Low | Unclear | High | Low | Low | High | 3 |
| Matarma (2020) | Unclear | High | High | Low | Low | High | 2 |
| McHale (2001) | Low | Low | Unclear | Low | Low | Low | 5 |
| Miranda-Rios (2017) | Low | Unclear | High | High | High | High | 1 |
| Moore (2020) | High | Low | High | Low | Low | High | 3 |
| Moran (2017) | Low | High | High | Low | Low | High | 3 |
| Mota (2017) | High | Low | Low | Low | Low | Low | 5 |
| Muthuri (2015) | Unclear | Low | Unclear | Low | Low | High | 3 |
| Nicksic (2018) | Low | Low | High | Low | Low | Low | 5 |
| Nordvall-Lassen (2018) | Low | Low | Unclear | High | Low | Low | 4 |
| Nystrom (2019) | Low | Low | Low | Low | Low | Low | 6 |
| Page (2010) | Low | Low | Low | Low | Low | Low | 6 |
| Predy (2020) | Low | Low | Low | High | Low | Low | 5 |
| Puett (2019) | Low | Low | Low | High | High | High | 3 |
| Ramírez-Izcoa (2017) | Unclear | Unclear | Unclear | Low | Low | High | 2 |
| Reimers (2017) | Unclear | Low | Low | Low | Low | High | 4 |
| Reimers (2018) | Low | Unclear | Low | Unclear | Low | High | 3 |
| Reimers (2019) | Low | High | High | Low | Low | Low | 4 |
| Remmers, Broeren (2014) | Low | Low | Unclear | High | Low | Low | 4 |
| Remmers, Kann (2014) | Low | Low | Unclear | High | Low | Low | 4 |
| Riiser (2019) | Low | Low | Low | Low | Low | High | 5 |
| Schoeppe (2017) | High | High | Low | Low | Low | Low | 4 |
| Sharp (2018) | High | High | High | Low | Low | Low | 3 |
| Spurrier (2008) | Low | High | High | Low | Low | High | 3 |
| Stone (2014) | Low | Low | Unclear | Low | High | Low | 4 |
| Tandon (2012) | Low | High | High | Low | Low | Low | 4 |
| Tappe (2013) | Low | High | Low | Low | Low | Low | 5 |
| van Rossem (2012) | Low | Unclear | Unclear | Low | Low | Low | 4 |
| van Stralen (2012) | Low | Low | Low | Low | Low | Low | 6 |
| Vandewater (2007) | High | High | High | Unclear | High | Low | 1 |
| Veitch (2010) | Unclear | High | Low | Low | Low | Low | 4 |
| Villarreal-Calderon (2002) | Low | High | High | Low | High | High | 2 |
| Wang (2018) | Low | High | High | Low | Low | High | 3 |
| Weir (2006) | Low | High | High | High | Low | High | 2 |
| Wen (2009) | Unclear | High | Unclear | High | Low | High | 1 |
| Wijtzes (2014) | Low | High | Unclear | High | Low | Low | 3 |
| Wilkie (2018) | Unclear | High | Unclear | Low | Low | Low | 3 |
| Wiseman (2019) | High | Low | Low | Low | Low | Low | 5 |
| Xu (2016) | Unclear | High | High | Unclear | Low | Low | 2 |
| Xu (2017) | Unclear | High | Unclear | High | Low | Low | 2 |
| Yoon (2019) | Low | High | High | High | Low | Low | 3 |
| Zahl-Thanem (2019) | High | High | Low | High | Low | Low | 3 |
| Zhai (2018) | High | Unclear | High | High | Low | High | 1 |
